# The APC/C Coordinates Retinal Differentiation with G1 Arrest through the Nek2-Dependent Modulation of Wingless Signaling

**DOI:** 10.1016/j.devcel.2016.12.005

**Published:** 2017-01-09

**Authors:** Torcato Martins, Francesco Meghini, Francesca Florio, Yuu Kimata

**Affiliations:** 1 Cell Cycle Development Group, Department of Genetics, University of Cambridge, Cambridge CB2 3EH, UK

**Keywords:** APC/C, proteolysis, wingless, Wnt, Drosophila, Nek2, eye development, Dpp, differentiation, fate specification

## Abstract

The cell cycle is coordinated with differentiation during animal development. Here we report a cell-cycle-independent developmental role for a master cell-cycle regulator, the anaphase-promoting complex or cyclosome (APC/C), in the regulation of cell fate through modulation of Wingless (Wg) signaling. The APC/C controls both cell-cycle progression and postmitotic processes through ubiquitin-dependent proteolysis. Through an RNAi screen in the developing *Drosophila* eye, we found that partial APC/C inactivation severely inhibits retinal differentiation independently of cell-cycle defects. The differentiation inhibition coincides with hyperactivation of Wg signaling caused by the accumulation of a Wg modulator, *Drosophila* Nek2 (dNek2). The APC/C degrades dNek2 upon synchronous G1 arrest prior to differentiation, which allows retinal differentiation through local suppression of Wg signaling. We also provide evidence that decapentaplegic signaling may posttranslationally regulate this APC/C function. Thus, the APC/C coordinates cell-fate determination with the cell cycle through the modulation of developmental signaling pathways.

## Highlights

• APC/C inactivation disrupts retinal differentiation in the *Drosophila* eye
• APC/C inactivation causes the ectopic activation of Wg signaling
• APC/C^Fzr^ downregulates a Wg modulator, dNek2, by proteolysis upon G1 arrest
• Local dNek2 degradation ensures the coordination of retinal differentiation

## Graphical Abstract

**Figure:**
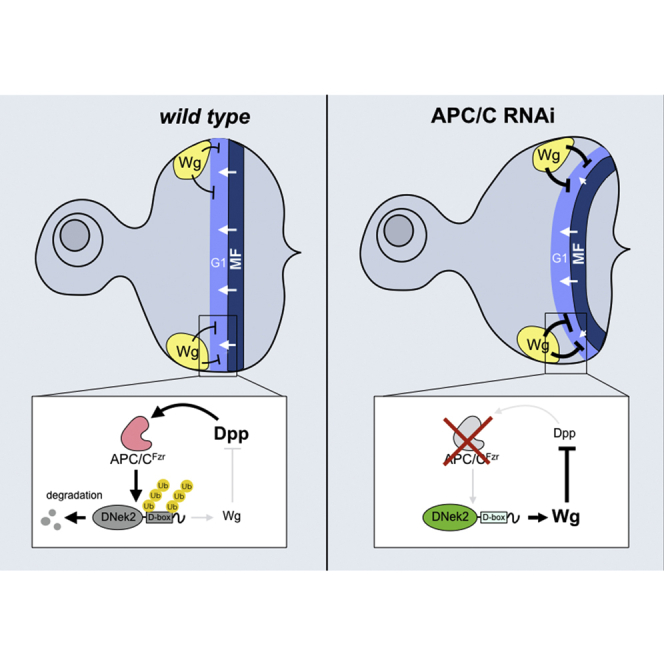

## Teaser

Martins et al. discovered a cell-cycle-independent function of the APC/C ubiquitin ligase in *Drosophila* eye development. They show that the APC/C controls retinal differentiation by locally suppressing Wingless signaling through the destruction of Nek2. Thus, the APC/C coordinates developmental signaling activity with the cell cycle.

## Introduction

During development of multicellular organisms, the cell cycle is highly coordinated with differentiation. After undergoing multiple rounds of the cell cycle to build the foundation of tissues and organs, the majority of cells eventually cease the cell cycle and initiate differentiation, except for a small number of progenitor cells that maintain the cell-cycle capacity and remain undifferentiated. Thus, the coordination between the cell cycle and differentiation must be strictly regulated to form and maintain functional tissues and organs. Uncoupling of this coordination may lead to tumorigenesis, tissue degeneration, and aging.

To facilitate this coordination, a set of the cell-cycle regulators possess the ability to regulate differentiation processes. Among such regulators is the anaphase-promoting complex or cyclosome (APC/C), an evolutionarily conserved ubiquitin ligase complex that controls cell-cycle progression via ubiquitin-mediated proteolysis (Pines, 2011). During mitosis, the APC/C binds the mitotic activator CDC20/Fizzy (Fzy) to drive chromatid separation and mitotic exit, whereas, during interphase, it interacts with the interphase activator CDH1/Fizzy-related (Fzr) to maintain G1 arrest or initiate the endocycle. Active in G1 phase, APC/C^Fzr^ plays a more prominent role in postmitotic APC/C functions and is involved in various cell-cycle-independent processes from metabolism and differentiation to neuronal activity (Eguren et al., 2011). Nevertheless, non-cell-cycle functions of the APC/C and the underlying mechanisms remain unexplored.

In the fruit fly *Drosophila melanogaster*, the eye primordium, called the eye imaginal disc, exhibits highly ordered patterns of proliferation and differentiation on a single epithelial cell sheet during the retinal differentiation processes (Baker, 2007, Kumar, 2011), providing an excellent in vivo model to study the coordination between the cell cycle and differentiation processes. The differentiation is coordinated by the posterior-to-anterior progression of the morphogenetic furrow (MF), in which cells are synchronously arrested in G1 phase and the first set of photoreceptor neurons are specified. A developmental role of the APC/C was first characterized in this model: a loss-of-function mutation in the APC/C activator *fzr* causes a failure in synchronous G1 arrest, leading to severe disruption of eye patterning (Karpilow et al., 1996). Furthermore, the mutations in *fzr* or *shattered* (*shtd*, *Drosophila* Apc1) result in ectopic mitosis of photoreceptor neurons (Ruggiero et al., 2012, Tanaka-Matakatsu et al., 2007). These findings underpin the role for the APC/C in two distinct cell-cycle processes: synchronized G1 arrest ahead of and within the MF and the maintenance of permanent G1 arrest in postmitotic neurons.

The coordinated movement of the MF and the cell-cycle state of the eye imaginal disc is controlled by the interplay between three conserved developmental signaling pathways. The hedgehog and decapentaplegic (Dpp, *Drosophila* BMP homolog) pathways initiate and promote the progression of the MF by inducing the synchronized G1 arrest ahead of the MF, whereas the Wingless (Wg, *Drosophila* Wnt) pathway inhibits MF progression and promotes cell proliferation in the anterior progenitor domain (Dominguez and Casares, 2005, Kumar, 2011). In addition, other signaling pathways, such as Notch and epidermal growth factor receptor, cooperate with these pathways to form the highly organized eye structure (Baker, 2007). The individual roles of each signaling pathway in eye development has been extensively studied. However, how these pathways cooperate with each other and regulate the cell cycle remains poorly understood.

In this study, we explored cell-cycle-independent functions of the APC/C in *Drosophila*. By performing an RNAi screen in the developing *Drosophila* eye, we found that partial APC/C inactivation strongly inhibits retinal differentiation. This phenotype is caused by hyperactivation of Wg signaling through stabilization of *Drosophila* NimA-related kinase 2 (dNek2). Our study demonstrates that the APC/C coordinates retinal differentiation by modulating Wg signaling through dNek2 degradation.

## Results

### Partial Depletion of APC/C Subunits Causes Defective Differentiation in the *Drosophila* Eye

To investigate a developmental function of the APC/C, we performed an in vivo RNAi screen against the subunits and activators of the APC/C in the *Drosophila* compound eye. We used two distinctive Gal4 driver lines to induce RNAi. *eyeless*-Gal4 (*ey-*Gal4) induces RNAi in the entire eye imaginal disc throughout its development, thereby affecting every step of differentiation and the growth of the eye disc. In contrast, glass multimer reporter-Gal4 (GMR-Gal4) induces RNAi only posterior to the MF in the eye disc, thereby affecting later differentiation processes without affecting the initial steps of photoreceptor differentiation.

It was shown previously that the hypomorphic *fzr* or *shtd* mutations cause roughening in the adult eye (Karpilow et al., 1996, Tanaka-Matakatsu et al., 2007). This “rough eye” phenotype is caused by ectopic mitosis of the photoreceptor neurons due to a loss of APC/C activity required for maintaining G1 arrest (Ruggiero et al., 2012). GMR-Gal4-driven *fzrRNAi*, *cdc27RNAi*, or *cdc23-likeRNAi* phenocopied the rough eye phenotype, validating our RNAi approach to inactivate the APC/C (Table S1, Figure S1A). The lack of phenotypes with other RNAi lines may be attributable to insufficient depletion of the targeted components due to their slow turn-over rate or a poor targeting capacity of the RNAi constructs. By the incubation at the higher temperature (29°C) or the co-expression of Dicer-2 (Dcr-2), a main component of the RNAi machinery, to increase the depletion efficiency, GMR-Gal4-driven *cdc16RNAi1* also showed the rough eye phenotype (Figure S1B).

**Figure 1:**
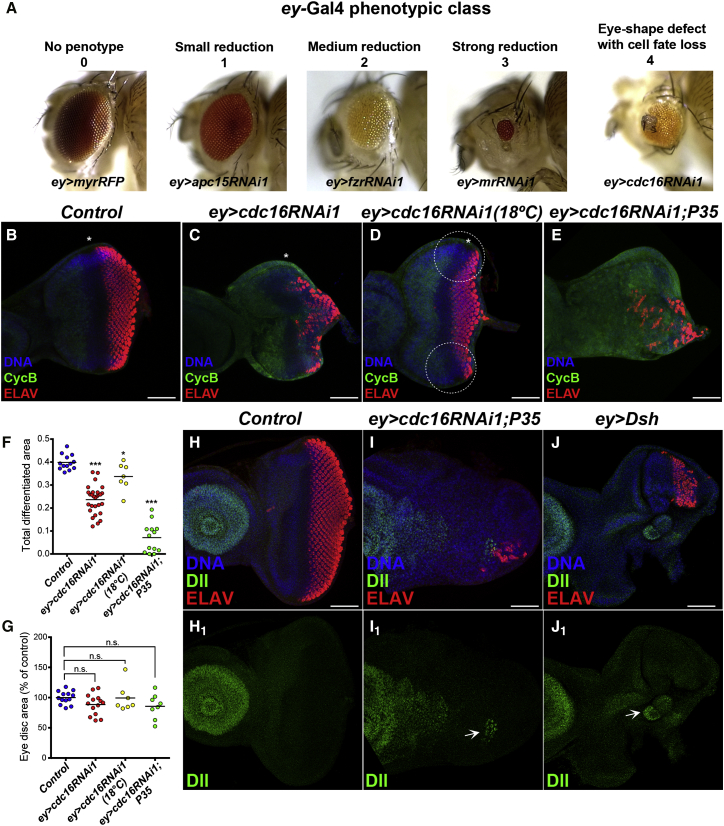
Partial Depletion of APC/C Subunits Causes the Inhibition of Retinal Differentiation (A) The examples of each category of the adult eye phenotypes observed upon *ey*-Gal4-driven induction of APC/C components: 0, no effect; 1, small reduction on retina size; 2, significant reduction in adult retinas; 3, strong eye reduction; 4, eye-shape defect with cell-fate loss. See also Figure S1 and Table S1. (B–E) Representative images of eye imaginal discs from third-instar larvae of the indicated genotypes, stained for DNA (blue), CycB (green), and a neuronal marker, ELAV (red). In control eye imaginal discs (B), the G1-arrested region (CycB-negative) precedes the uniform progression of photoreceptor differentiation (ELAV-positive). *ey>cdc16RNAI* at 25°C (C) or 18°C (D) results in the inhibition of MF progression, particularly evident in the lateral marginal regions (white circles) (D). Co-expression of P35 (E) enhanced the inhibitory effect of *ey>cdc16RNAi1* on retinal differentiation. Anterior to the left and posterior to the right. The MFs are indicated by asterisks. Scale bars, 50 μm. See also Figure S2. (F and G) The relative differentiated regions (ELAV-positive) (F) and the relative eye disc size (% of control) (G) of the indicated genotypes were measured and are shown in the dot plots. Each dot represents an individual measurement and horizontal bars indicate the mean values (n = 7–25, ^∗∗∗^p < 0.0001, ^∗^p < 0.05, n.s., no statistical difference between samples). (H–J_1_) Representative images of larval eye discs of the indicated genotypes, stained for DNA (blue), Dll (green), and ELAV (red). In control eye imaginal discs, Dll expression is restricted to the antenna domain of the eye-antenna imaginal disc (H, H_1_). Eye discs expressing P35 and *cdc16*RNAi (I, I_1_) or the Wg signaling component Dsh (J, J_1_) showed ectopic Dll mis-expression on the eye disc (white arrow) (I_1_, J_1_). Anterior to the left. Scale bars, 50 μm.

When *ey*-Gal4 was used, many of the RNAi lines caused size reduction of the adult eyes, as expected of the established role of the APC/C in cell proliferation (Figures 1A, S1C, and S1D, Table S1). *morulaRNAi1*, *idaRNAi3*, and *fzrRNAi1* showed the strongest effect on eye size, in conjunction with high lethality (class 3 in Figure 1A, Table S1). In contrast, the *cdc16RNAi* lines, *idaRNAi1* and *shtdRNAi1*, only mildly affected the retinal size, but severely disrupted the morphology and polarity of the adult eye (class 4 in Figures 1A, S1D, and S1E, Table S1). Furthermore, these flies frequently formed ectopic structures within the eye field that resemble naked head cuticles or antennae, indicative of a complete loss or a conversion of cell fate (52.01% ± 2.04% head-like structure, 23.12% ± 3.76% antenna-like structure, n = 6, Figures 1A and S1C). Similar ectopic structures were observed previously upon ectopic activation of Wg signaling in the larval eye imaginal disc (Baonza and Freeman, 2002). We were able to reproduce this eye phenotype by expressing a Wg pathway component, Disheveled (Dsh), by *ey-*Gal4 (66.29% ± 6.65% head-like structure, 29.57% ± 6.72% antenna-like structure, n = 6, Figure S1F). To our knowledge, this eye phenotype (hereafter, referred to as “differentiation failure” phenotype) has never been reported for any APC/C mutants. This phenotype is attributable to partial inactivation of the APC/C (a reduction in its catalytic activity or a loss of its ability to target a specific substrate), which is achieved by partial depletion of the individual subunits. The co-expression of Dcr-2 converted the phenotype of *ey>cdc16RNAi1* to a strong reduction in the eye size (class 3 in Figure 1). Thus, more complete depletion of the APC/C subunits causes mitotic arrest, leading to eye size reduction, by blocking the cell-cycle function of the APC/C.

### APC/C Inactivation Disrupts the Uniform Progression of the MF in the Eye Imaginal Disc

During the third-instar larval (L3) stage, the MF sweeps through the eye imaginal disc from the posterior tip to the anterior end (Baker, 2007). *ey-*Gal4-driven *cdc16RNAi1* (hereinafter, *ey>cdc16RNAi1*) strongly inhibits retinal differentiation in the eye disc (Figures 1B, 1C, and 1F) without affecting the size of the eye disc (Figure 1G). While the synchronized G1 arrest is maintained in the majority of the eye discs (asterisk, Figures 1B–1D), the progression of the MF is severely inhibited by *ey>cdc16RNAi1* (Figures 1B and 1C). Importantly, when the induction level of *ey>cdc16RNAi1* was reduced at 18°C, the delay in MF progression was more evident toward the lateral margins compared with the medial region of the eye disc (white circles, Figure 1D), suggesting that *ey>cdc16RNAi1* primarily affects MF progression in the lateral marginal regions. We also observed a similar delay in MF progression, using *shtdRNAi1* and *idaRNAi1*, as well as *cdc16RNAi3*, which targets a different region of the *cdc16* gene (Figures S2G–S2J). Moreover, both the differentiation failure in the adult eye and the inhibition of MF progression caused by *ey>cdc16RNAi1* was rescued by the expression of the *pUbq-GFP-Cdc16* transgene (Figures S2K and S2L). These results indicate that the APC/C is required for the coordinated progression of retinal differentiation in the eye disc. We primarily utilize *cdc16RNAi1* in further analyses of these phenotypes.

### APC/C Inactivation Inhibits Retinal Differentiation Independently of Cell-Cycle Defects and Apoptosis

Since *ey*-Gal4 induces RNAi throughout the eye imaginal disc development, it is possible that the differentiation failure may be a secondary consequence of cell-cycle arrest or undergrowth of the eye disc caused by APC/C inactivation. *ey>cdc16RNAi1* did not significantly affect the overall size of the eye disc (Figures 1B–1D). Although *ey>cdc16RNAi1* mildly increased the number of mitotic cells in the eye disc (mitotic index) (Figures S2A, S2B, and S2F), there is no direct correlation between the increase in the mitotic index and the degree of differentiation inhibition: co-expression of the apoptotic inhibitor P35 significantly enhanced the inhibitory effect of *ey>cdc16RNAi1* on retinal differentiation (Figures 1E, 1F, and S1G), but not the mitotic index (Figures S2D–S2F). Furthermore, when induced at 18°C, *ey>cdc16RNAi1* inhibited MF progression in the lateral margins without affecting the mitotic index (Figures 1D, S2C, and S2F). We also tested genetic interactions with the cell-cycle regulator, *cyclin A* (*cycA*). In alignment with a previous report (Kaplow et al., 2007), *fzrRNAi1* partially rescued the strong eye size reduction of *ey*>*cycARNAi* (Figure S2M), whereas *cdc16RNAi1* did not affect the phenotype (Figure S2N). These results strongly suggest that the differentiation failure phenotype induced by *ey>cdc16RNAi1* is separable from cell-cycle defects.

It was previously shown that in the eye discs of the *fzr* and *shtd* mutants many cells are eliminated by apoptosis (Karpilow et al., 1996, Tanaka-Matakatsu et al., 2007). To examine the possible involvement of apoptosis, we inhibited apoptosis by co-expressing P35, or using Def(3L)H99, the deficiency mutation that lacks several major apoptotic genes (White et al., 1994). In both cases, the apoptosis inhibition enhanced the differentiation failure phenotype of *ey>cdc16RNAi1*, resulting in the formation of disorganized undifferentiated masses in the adult eye field (Figure S1G). P35 co-expression enhanced the differentiation inhibition of *ey>cdc16RNAi1* in the eye discs (Figures 1E–1G), forming many folds, uncharacteristic of this tissue (Movies S1, S2, and S3), and also caused mis-expression of Distal-less, the fate marker for ventral appendages including antennae in the eye disc, indicating cell-fate conversion (Figures 1H–1I). In alignment with the previous report (Baonza and Freeman, 2002), *ey>Dsh* also caused Dll expression (Figure 1J). Thus, apoptosis does not contribute to the differentiation failure phenotype of *ey>cdc16RNAi1*, but, instead, suppresses further aggravation.

### APC/C Inactivation Induces Ectopic Activation of Wg Signaling

The progression of the MF involves two separate processes: first, the formation of the MF at the posterior tip of the eye disc; second, the repeated induction of MF formation at the lateral eye margins, termed “reincarnation,” which ensures the uniform progression of the MF across the dorsoventral axis (Chanut and Heberlein, 1997, Kumar and Moses, 2001, Pignoni and Zipursky, 1997, Wiersdorff et al., 1996). The presence of the MF at the posterior medial region (Figure 1C) and the stronger inhibition on MF progression in the lateral margins in *ey>cdc16RNAi1* eye discs (Figure 1D) suggests that APC/C inactivation is likely to mainly impact on the reincarnation process by attenuating the differentiation-promoting activity of Dpp signaling, or by upregulating the inhibitory activity of Wg signaling that originates from the antero-lateral marginal regions.

**Figure 2:**
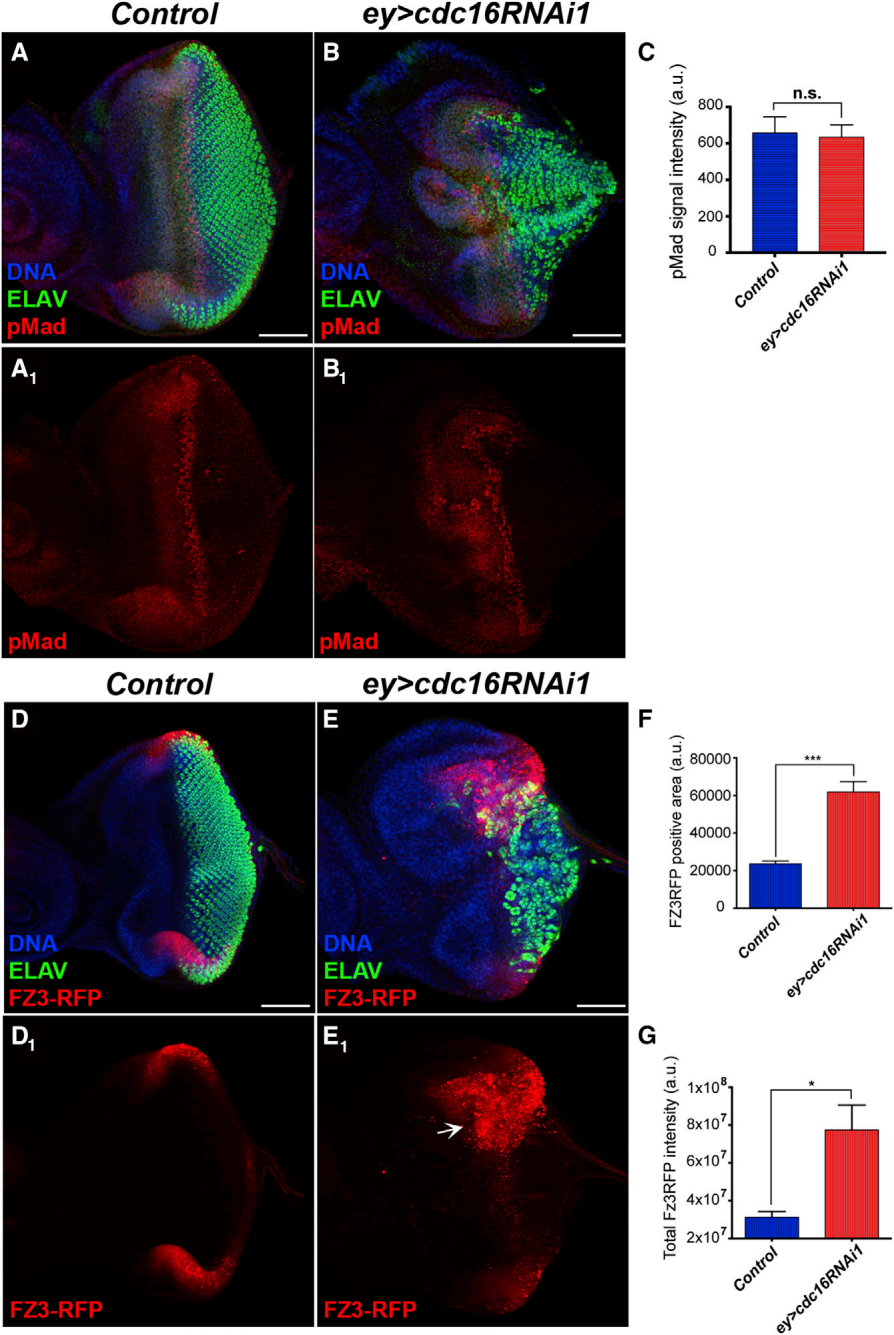
Cdc16 Depletion Promotes Ectopic Activation of Wg Signaling (A–B_1_) Eye imaginal discs of *ey-*Gal4 (control) (A) and *ey>cdc16*RNAi1 (B) stained for DNA (blue), ELAV (green), and pMad (red). Despite the severe differentiation delay, the levels of pMad were not significantly affected by *ey>cdc16RNAi* (B_1_) compared with the control (A_1_). Posterior to the right. Scale bars, 50 μm. See also Figure S3. (C) The total intensities of the pMad signals in the control and *ey>cdc16*RNAi eye discs were measured and the means are represented in a bar graph (n = 12, n.s., no statistical difference, the error bars indicate the SD). (D–E_1_) Eye imaginal discs of *ey-*Gal4 (control) (D, D_1_) and *ey>cdc16*RNAi1 (E, E_1_) carrying the Wg reporter Fz3-RFP (red), stained for DNA (blue) and ELAV (green). *ey>cdc16*RNAi1 induced the accumulation of Fz3-RFP signals at the eye lateral margins (white arrow) as well as central regions of the eye field (E_1_). Scale bars, 50 μm. (F and G) The Fz3-RFP-positive areas (F) and the signal intensities (G) in the control and *ey>cdc16*RNAi eye discs were measured and the mean values are shown in bar graphs. The error bars indicate the SEM (n = 10–12, ^∗^p < 0.05, ^∗∗∗^p < 0.0001).

To test this hypothesis, we examined the effect of *ey>cdc16RNAi1* on the activity of these two signaling pathways. We first analyzed Dpp signaling activity by using an enhancer trap line for Dpp (Dpp-lacZ) and an antibody that recognizes the phosphorylated form of *Drosophila* Smad2/3, Mad (pMad). In unperturbed eye imaginal discs, both lacZ and pMad signals were detected at the MF and in the lateral disc margins (Figures 2A and S3A). There was no significant effect of *ey>cdc16RNAi1* on the intensity of pMad signals (Figures 2A–2C). Dpp-lacZ enhanced the differentiation inhibition of *ey>cdc16RNAi1*, leading to no MF formation, which is likely to be due to the dilution of the Dpp auto-activation (Chanut and Heberlein, 1997) caused by an extra non-functional copy of the Dpp regulatory region in the Dpp-lacZ reporter (Figures S3A–S3B). Nevertheless, *ey>cdc16RNAi1* did not affect either the total intensity or the area of Dpp-lacZ signals (Figures S3A–S3D). *ey>cdc16RNAi1* also did not affect the expression of a Notch reporter, NRE-GFP (Zacharioudaki and Bray, 2014) (Figures S3E–S3F).

We next analyzed Wg signaling activity by using the reporter construct for the transcriptional level of a positive Wg target, Frizzled 3 (Fz3-RFP) (Sato et al., 1999). In control eye discs, the Fz3-RFP reporter exhibited strong signals at the lateral disc margins, recapitulating the pattern of endogenous Wg signaling activity (Treisman and Rubin, 1995) (Figure 2D). *ey>cdc16RNAi1* caused a massive expansion of Fz3-RFP signals and their significant accumulation around the lateral margins, where a delay in retinal differentiation was particularly evident (Figures 2E–2G). These results strongly suggest that APC/C inactivation causes the upregulation of the Wg pathway, leading to the inhibition of retinal differentiation.

### *Drosophila* Nek2 Is an APC/C^Fzr^ Substrate

A recent overexpression screen using the expression library of *Drosophila* genes (FlyORF) uncovered an unexpected activity of dNek2 to upregulate Wg signaling activity (Schertel et al., 2013). We have previously shown that human Nek2 is an APC/C substrate (Hayes et al., 2006). Therefore, we hypothesized that dNek2 might mediate the Wg signaling upregulation induced by APC/C inactivation.

**Figure 3:**
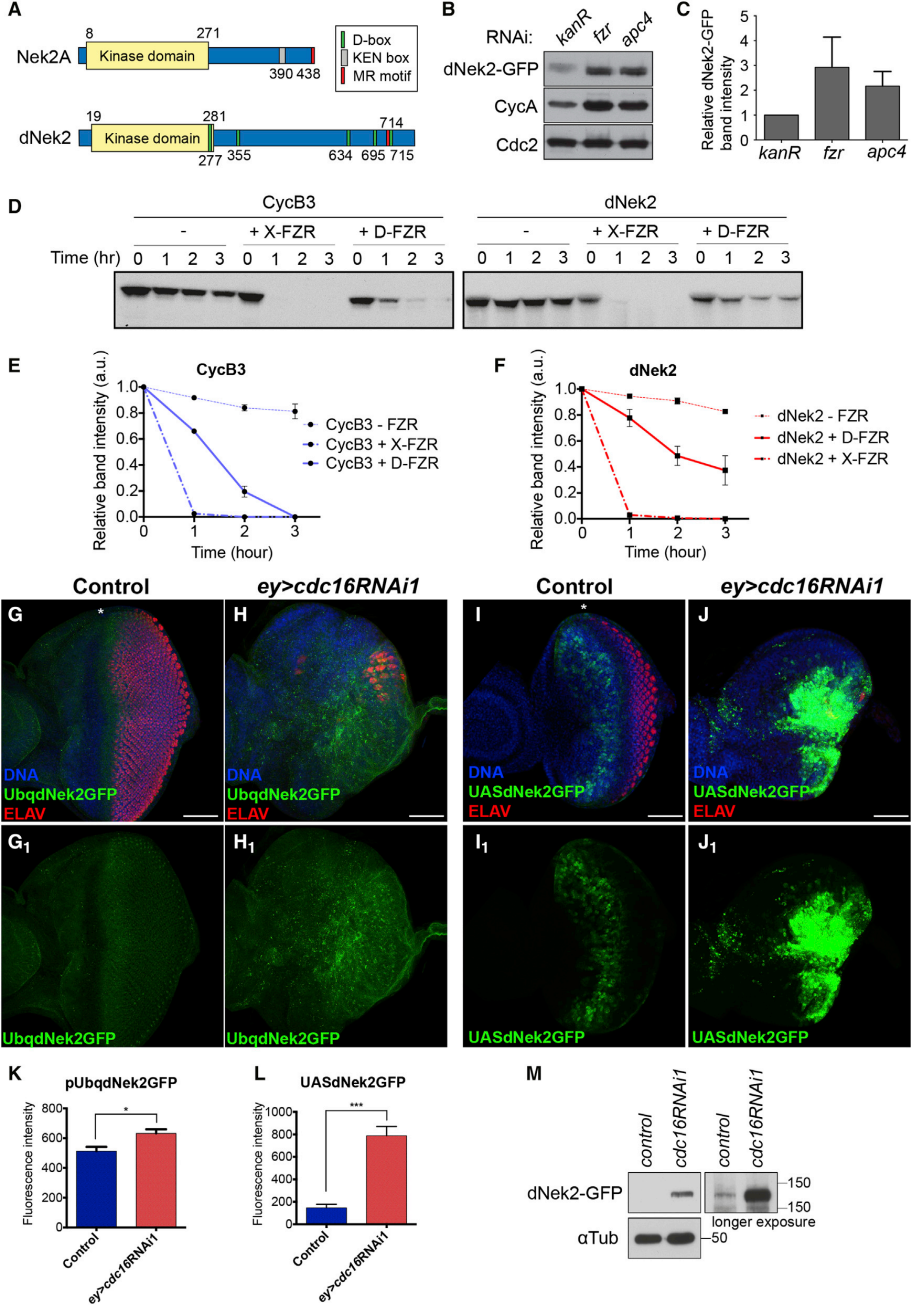
dNek2 Is An APC/C^Fzr^ Substrate (A) Schematic representations of human Nek2A and dNek2 proteins: Nek2A has two functional APC/C recognition motifs, KEN box (gray box) and MR motif (red box). dNek2 has five D box-like motifs (green boxes) and one MR motif (red box). (B and C) Immunoblotting of *D.mel-2* cell lysates stably expressing dNek2-GFP. RNAi of *fzr* or *apc4*, but not *kanR*, accumulated dNek2-GFP and the canonical APC/C substrate, CycA. dNek2-GFP band intensities (relative to *kanR*) were quantified and the mean values are presented in a bar graph (C) (n = 3, error bars represent SEM). (D–F) In vitro destruction assays of CycB3 and dNek2, labeled with sulfur-35, in interphase extracts in the presence or absence of the recombinant *Xenopus* Fzr (X-Fzr) or *Drosophila* Fzr (D-Fzr). The samples were analyzed in autoradiographs (D). Similar to CycB3 (D) (left panel) dNek2 was efficiently degraded upon the addition of X-Fzr or D-Fzr (D) (right panel). The relative CycB3 (E) or dNek2 (F) band intensities (relative to time 0) were quantified and the mean values are presented in line graphs (n = 3, error bars represent SEM). See also Figure S4. (G–L) The control (*ey-*Gal4) and *ey>cdc16*RNAi1 eye discs carrying *pUbq-dNek2-GFP* (G–H_1_) (green) or *UAS-dNek2-GFP* (I–J_1_) (green), stained for DNA (blue) and ELAV (red). Anterior to the left. Weak dNek2-GFP signals (green) were detected in both the anterior and posterior regions to the MF in the control carrying *pUbq-dNek2-GFP* (G, G_1_) and *ey>cdc16*RNAi accumulated dNek2-GFP in the posterior region of the eye imaginal discs (H, H_1_). In the control eye disc inducing dNek2-GFP by *ey-Gal4* (control) (I, I_1_), dNek2-GFP signals were detected anteriorly to the MF, but absent in the posterior region in the early L3 stage. *ey>cdc16*RNAi caused robust accumulation of dNek2-GFP signals in the posterior region of the eye discs (J, J_1_). Asterisks mark the MF. Scale bars, 50 μm. dNek2-GFP signal intensities were quantified and their mean values are presented in bar graphs, (K) and (L), respectively. Error bars indicate SEM (n = 8–12, ^∗∗∗^p < 0.0001, ^∗^p < 0.05). (M) Immunoblot analysis of eye-antenna disc extracts of the control (*ey-*Gal4) or *ey>cdc16RNAi1* carrying *UAS-dNek2-GFP*. dNek2-GFP was accumulated upon *cdc16*RNAi. α-Tubulin was used as a loading control.

dNek2 and human Nek2 proteins show a high amino acid sequence similarity in the catalytic domain, but are quite diverged in the non-catalytic C-terminal domains (Figure 3A). We found that GFP-tagged dNek2 (dNek2-GFP), as well as a canonical APC/C substrate, CycA, significantly accumulated upon the depletion of endogenous Fzr or Apc4 by RNAi in *D.mel-2* cultured cells (Figures 3B and 3C). To examine whether the APC/C can directly target dNek2 for degradation, we performed in vitro reconstituted APC/C-dependent destruction assays using *Xenopus laevis* egg extracts. In mitotic extracts that contain active APC/C^Fzy^, dNek2 did not show any sign of degradation, although a canonical APC/C substrate, cyclin B3 (CycB3), was efficiently degraded in the absence of the APC/C inhibitor Mes1 (Figures S4A and S4B). In contrast, in interphase extracts, dNek2 was rapidly degraded upon the addition of recombinant *Xenopus* or *Drosophila* Fzr (X-Fzr or D-Fzr, Figures 3D–3F), establishing dNek2 as an APC/C^Fzr^ substrate.

We next examined whether dNek2 is targeted by APC/C for degradation in vivo. To detect dNek2, we generated two different dNek2-GFP transgenes, one containing GFP-tagged dNek2 under the control of constitutively active poly-ubiquitin promoter (*pUbq-dNek2-GFP*) and the other containing dNek2-GFP under the Gal4-inducible upstream activation sequence (UAS) element (*UAS-dNek2-GFP*). In the control eye discs, *pUbq-dNek2-GFP* showed weak GFP signals throughout the eye disc apart from the MF region, where the GFP signals were clearly reduced (Figure 3G). *ey*-Gal4-driven *UAS-dNek2-GFP* showed strong dNek2-GFP signals in the anterior regions to the MF in the early L3 stage (Figure 3I). However, the dNek2-GFP signals disappeared to become virtually undetectable in the late L3 stage (data not shown). Upon *ey>cdc16RNAi1* induction, both *dNek2-GFP* transgenes showed a significant accumulation of dNek2-GFP in the posterior region of the eye disc concomitantly with strong differentiation inhibition (Figure 3H and 3J–3L). Consistently, by western blotting analysis, we also observed a significant increase in the dNek2-GFP levels in the lysates from the eye-antenna discs expressing *dNek2-GFP* upon *ey>cdc16RNAi1* induction (Figure 3M). These results have demonstrated that dNek2 is also degraded by the APC/C in vivo and is accumulated in the eye discs upon *ey>cdc16RNAi1*.

### dNek2 Mediates the Differentiation Failure Phenotype Caused by APC/C Inactivation

**Figure 4:**
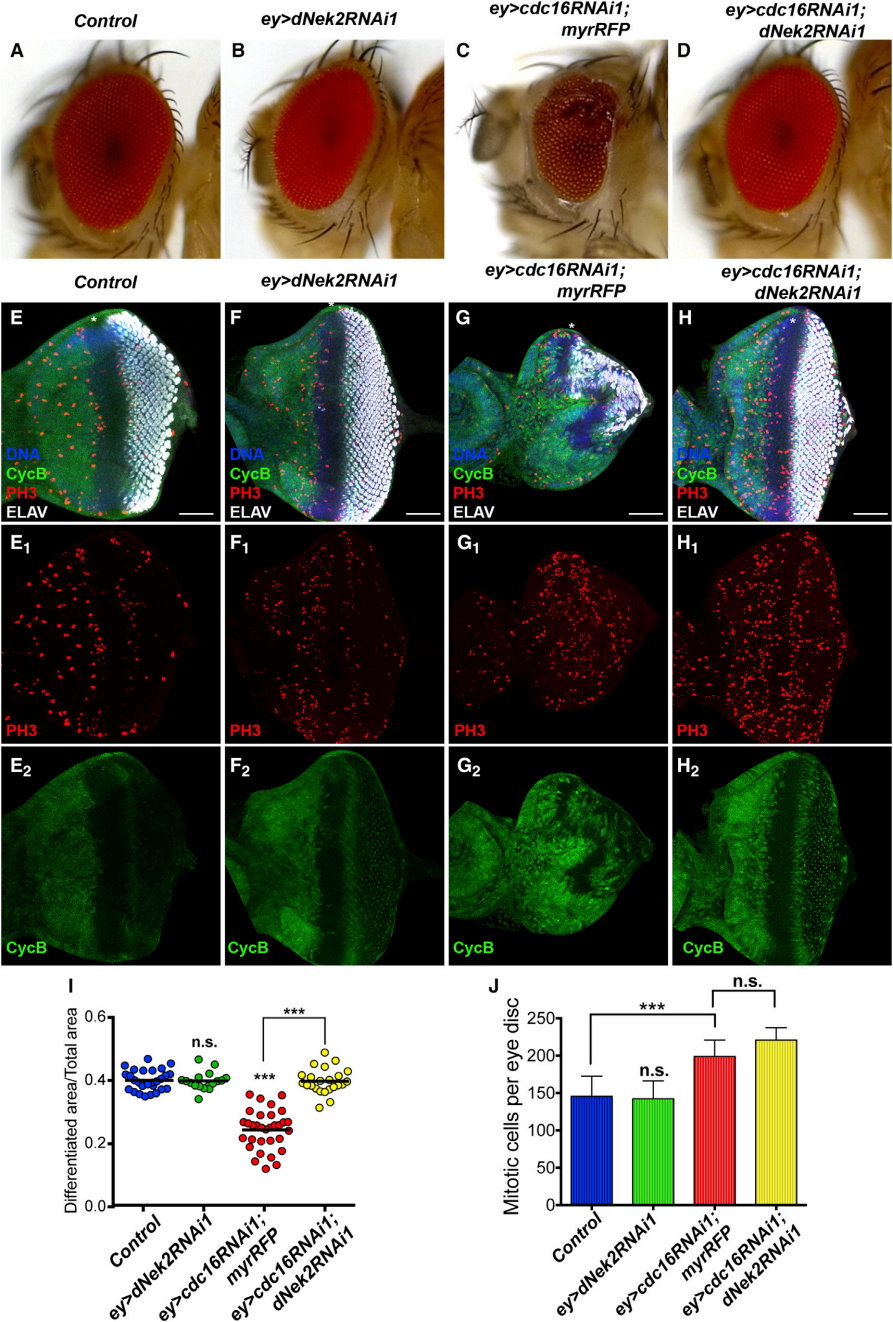
dNek2 Mediates the Differentiation Impairment Caused by Cdc16 Depletion (A–D) Adult eyes of the indicated genotypes. *ey>dNek2RNAi1* did not show any detectable defects (B). However, it fully rescued the differentiation failure phenotype of *ey>cdc16RNAi1* (C) and (D). (E–J) Eye imaginal discs of the indicated genotypes stained for DNA (blue), CycB (green), PH3 (red), and ELAV (white). Anterior to the left. The eye discs of *ey>dNek2RNAi* (F–F_2_) are comparable with the control (E–E_2_). However, *dNek2RNAi1* fully restored photoreceptor differentiation and the uniform MF progression in the eye discs of *ey>cdc16RNAi1* (H–H_2_) compared with (G–G_2_), without restoring the increased number of mitotic cells (G_1_, H_1_). Scale bars, 50 μm. Relative differentiated regions were quantified and are presented in a scattered dot plot (I). Each dot represents an individual measurement and horizontal bars the mean values (n = 15–30). The numbers of mitotic cells per eye disc were quantified and the mean values are presented in a bar graph (J). The error bars indicate SD (n = 6–22). ^∗^p < 0.05, ^∗∗∗^p < 0.0001. n.s., no statistical difference. See also Figure S5.

We next addressed whether the differentiation failure phenotype upon *ey>cdc16RNAi* is mediated by dNek2 stabilization. When induced by *ey*-Gal4, neither of the *dNek2RNAi* constructs (*dNek2RNAi1* and *dNek2RNAi2*) showed any obvious effect on the eye imaginal disc or the adult eye (Figures 4A, 4B, 4E, 4F, 4I, 4J, and S5A). However, when co-induced with *cdc16RNAi1* by *ey-*Gal4, both *dNek2RNAi1* and *dNek2RNAi2* fully rescued the differentiation failure phenotype in the adult eye (Figures 4C, 4D, and S5A) and also restored the delay in retinal differentiation and the disorganization of MF progression in the eye disc (Figures 4G–4I). It is noteworthy that, despite its ability to fully rescue retinal differentiation defects, *dNek2RNAi1* did not rescue the mitotic index increase caused by *ey>cdc16RNAi1* (Figures 4G, 4H, and 4J). These results suggest that dNek2 degradation mediates the function of the APC/C in the regulation of retinal differentiation, but not its function in cell-cycle control.

To further assess the importance of dNek2 degradation for the regulation of retinal differentiation, we examined whether there is any synergistic effect between APC/C inactivation and overexpression of dNek2. *pUbq-dNek2-GFP* or *UAS-dNek2-GFP* both aggravated the differentiation failure phenotype caused by *ey>cdc16RNAi* in the adult eye (Figure S5B). To exclude a potential dominant-negative effect of the GFP tagging we also used an untagged *dNek2* transgene, described in the aforementioned screen (Schertel et al., 2013). *ey*-Gal4-driven expression of untagged dNek2 alone had no clear impact upon MF progression or adult eye morphology (Figures S5B, S5F, and S5G). However, the expression of untagged dNek2 significantly enhanced the differentiation defect induced by *ey>cdc16RNAi1* in the eye imaginal disc (Figures S5C–S5G) as well as the differentiation failure phenotype in the adult eye, even further than dNek2-GFP, leading to a massive expansion of the head case-like structure (Figure S5B). Collectively, these results strongly suggest that the expression levels of dNek2 are under the strict control of the APC/C to limit Wg signaling activity for the coordinated progression of retinal differentiation.

### The APC/C Downregulates dNek2 via Proteolysis upon Synchronized G1 Arrest

**Figure 5:**
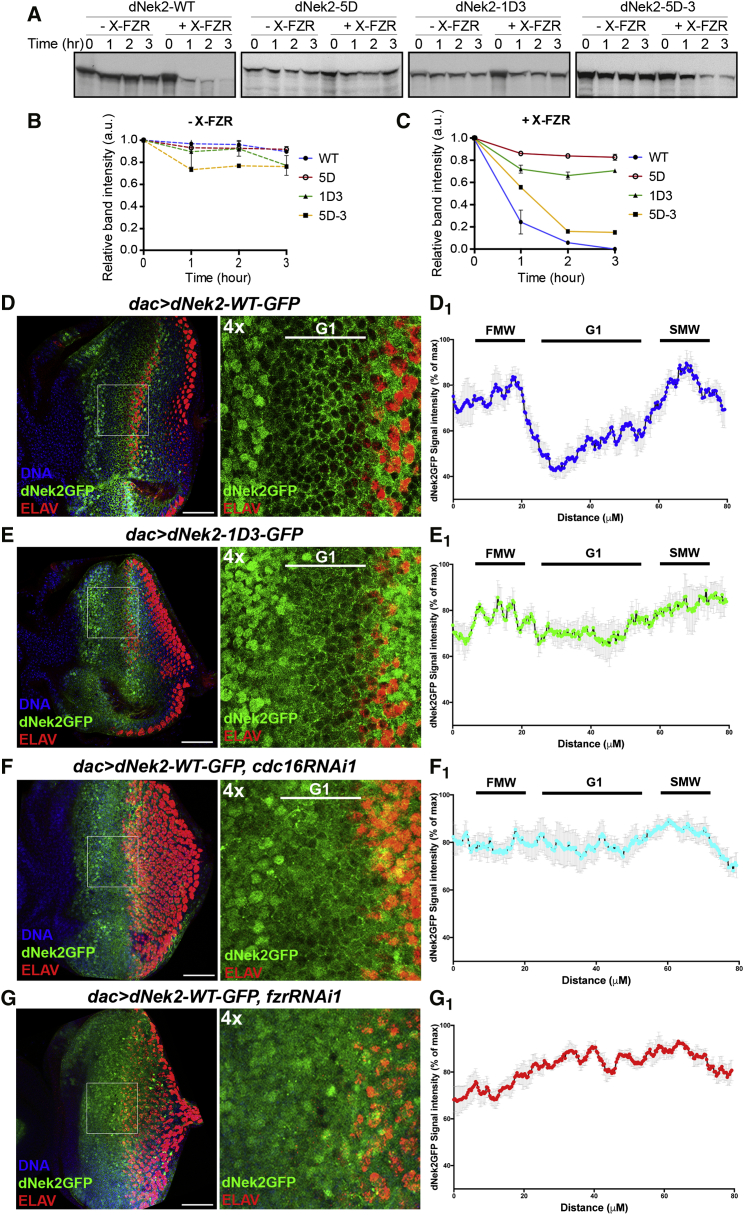
APC/C^Fzr^ Downregulates dNek2 via Degradation in the G1-Arrested Region of the Eye Disc (A–C) In vitro destruction assays in interphase egg extracts using the wild-type (dNek2-WT) and the D box mutants (dNek2-5D, dNek2-1D3, and dNek2-5D-3) of dNek2 labeled with sulfur-35. The samples were analyzed in autoradiographs (A). Both dNek2-5D and dNek2-1D3 were stable in the presence of X-FZR. The reversion of the third D box mutation on dNek2-5D (dNek2-5D-3) restored APC/C-dependent degradation. The dNek2 band intensities (relative to time 0) in the destruction assays in the absence (-X-Fzr) (B) or presence of X-Fzr (+X-Fzr) (C) were quantified and are shown in line graphs (n = 3, error bars indicate SEM). See also Figure S6. (D–G) Eye imaginal discs inducing the indicated *dNek2-GFP* constructs in the G1-arrested region by *dac*-Gal4, stained for DAPI (blue) and ELAV (red). The right panels correspond to 4× magnifications of the white squares drawn on the left panels. Anterior to the left. Scale bars, 50 μm. The dNek2-GFP signal intensities along the posterior-anterior axis in the MF regions were quantified and the mean values are plotted in the line graphs (D_1_–G_1_). Error bars correspond to SEM (n = 4–5). dNek2-GFP-WT levels are high at the first mitotic wave (FMW), decrease in the G1-arrested region, and re-accumulate at the second mitotic wave (SMW) (D, D_1_). dNek2-1D3-GFP was stabilized in the G1-arrested region (E, E_1_). *cdc16RNAi1* also stabilized dNek2-WT-GFP in the G1-arrested region (F, F_1_). *fzrRNAi1* disrupted both the synchronized G1 arrest (G) and the drop of dNek2-GFP (G, G_1_).

We next addressed how dNek2 levels are spatiotemporally regulated by the APC/C in the eye disc and whether this regulation plays a critical role in the uniform movement of the MF. We first sought to identify the motif responsible for the APC/C-dependent degradation of in dNek2. Human Nek2A is recognized by the APC/C via a canonical APC/C degron, the KEN box (at position 390), and a unique motif, the MR motif, situated at its C-terminal end (Figure 3A). dNek2 contains several putative APC/C degrons: five D box-like motifs (RxxL) at position 277, 355, 634, 695, and 715, and one MR motif at position 714 (Figure 3A). To determine which motif is required for dNek2 destruction, the D box motifs and the MR motif were mutated to AxxA and AA, respectively. The resultant dNek2 mutant proteins, dNek2-5D and dNek2-MR, were used in the in vitro APC/C-dependent degradation assays. dNek2-5D was completely stable in interphase extracts, whereas dNek2-MR was degraded upon the addition of X-Fzr at a comparable rate with wild-type dNek2 (dNek2-WT, Figures S6A–S6C). The kinase dead mutation K48M (Schertel et al., 2013) did not affect dNek2 degradation, indicating that the kinase activity is dispensable for APC/C^Fzr^-dependent degradation of dNek2 (Figures S6A–S6C). We then mutated each of the five D box motifs to generate single D box mutant proteins (dNek2-1D1 to 1D5). We found that the mutation of the third D box at position 355 (dNek2-1D3) significantly stabilized dNek2 in interphase extracts (Figures 5A–5C), whereas the mutations in the other D box motifs had little effect on its stability (Figures S6D–S6F). Moreover, the reversion of the third D box mutation in the dNek2-5D mutant (dNek2-5D-3) destabilized dNek2-5D in interphase extracts (Figures 5A–5C). These results have identified the third D box motif as the APC/C degron required and sufficient for APC/C^Fzr^-dependent degradation of dNek2.

To determine the regulation of APC/C-dependent degradation of dNek2 in vivo, we generated the transgenes that express GFP-fused, APC/C-undegradable, dNek2-1D3 (dNek2-1D3-GFP). In comparison with *pUbq-dNek2-WT-GFP*, which showed weak GFP signals throughout the eye disc apart from a clear reduction around the MF region (Figures 3G and S6G), *pUbq-dNek2-1D3-GFP* exhibited somewhat higher GFP signals in the posterior side of the eye disc. This accumulation of dNek2-1D3-GFP starts from the region immediately anterior to the MF where cells are synchronously arrested in G1 phase in response to Dpp signaling (Figure S6H). To investigate the regulation of dNek2 levels in this G1-arrested region in more detail, we used *dachshund*-Gal4 (*dac*-Gal4) to induce dNek2-GFP specifically in the vicinity of the MF that includes the first mitotic wave (FMW), the G1-arrested region and the second mitotic wave (SMW), but not the anterior progenitor domain (Tavsanli et al., 2004). When induced by *dac*-Gal4, dNek2-WT-GFP highly accumulated in the cells in the FMW and SMW regions, exhibiting uniform subcellular distribution throughout the cells (Figure 5D). However, in the G1-arrested region between the two mitotic domains, dNek2-WT-GFP signals were significantly lower in the cytoplasm, with clear GFP signals only at the intercellular junctions (Figure 5D). We found that APC/C-undegradable dNek2-1D3-GFP retains much higher cytoplasmic signals in the G1-arrested region in comparison with dNek2-WT-GFP (Figure 5E), suggesting that the APC/C degrades the cytoplasmic pool of dNek2 specifically in the G1-arrested region. Consistently, the induction of *cdc16RNAi1* by *dac*-Gal4 also accumulated dNek2-WT-GFP within the cells in this region (Figure 5F). *dac*-Gal4-driven *fzrRNAi1* completely abolished the downregulation of dNek2-WT-GFP (Figure 5G), disrupting G1 arrest (Pimentel and Venkatesh, 2005). Taken together, these results have demonstrated that APC/C^Fzr^ downregulates dNek2 levels via degradation specifically in the G1-arrested region ahead of and within the MF in the eye disc.

### The APC/C Facilitates the Uniform MF Progression by Downregulating dNek2 in the G1-Arrested Region

**Figure 6:**
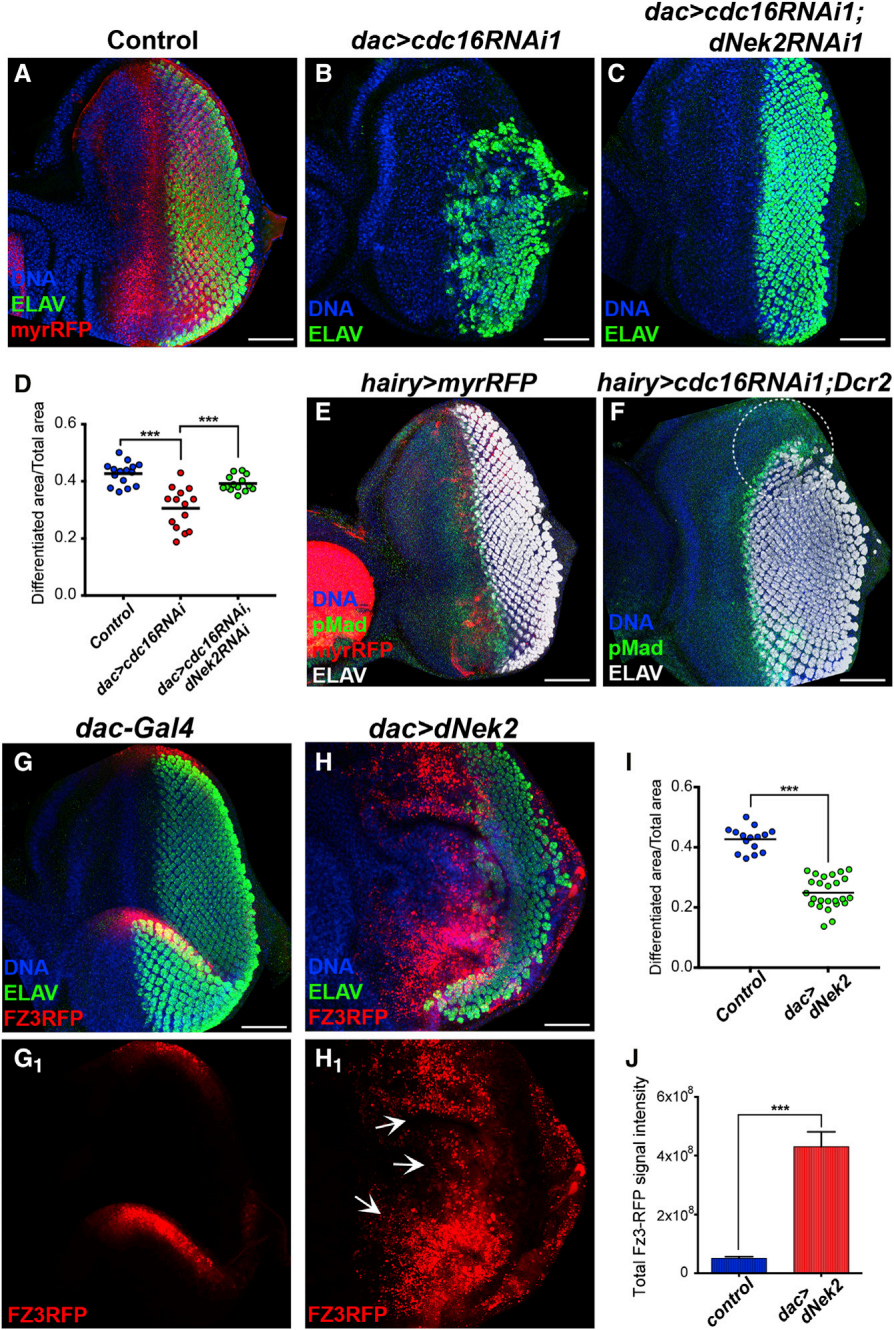
The G1-Arrest-Specific Degradation of dNek2 Promotes Uniform MF Progression and Suppresses Ectopic Wg Activation (A–D) Eye imaginal discs of the indicated genotypes stained for DNA (blue) and ELAV (green). In the control (*dac>myrRFP*), *dac*-Gal4 expressed myr-RFP (red) in the domain that spans from the FMW to the SMW (A). *dac*>*cdc16RNAi1* severely inhibited retinal differentiation in the lateral marginal regions (B), which was restored by co-induction *of dNek2RNAi1* (C). Anterior to the left. Scale bars, 50 μm. Relative differentiated regions were quantified and are shown in a dot plot (D). Each dot represents an individual measurement and horizontal bars the mean values (n = 14–20, ^∗∗∗^p < 0.0001). (E and F) Eye discs of *hairy>myrRFP* (control) (E) and *hairy>cdc16RNAi1*;*Dcr-2* (F) stained for DNA (blue), pMad (green), and ELAV (white). In the control, *hairy*-Gal4 expressed myrRFP (red) in a short and transient domain within the G1-arrested region (E). *hairy>cdc16RNAi* caused a delay in retinal differentiation in the lateral marginal regions (dotted white circle) (F). Anterior to the left. Scale bars, 50 μm. (G–J) Eye imaginal discs carrying the Wg reporter Fz3-RFP (red), stained for DNA (blue), and ELAV (green). The control (*dac-*Gal4) showed Fz3-RFP signals at the lateral margins (G, G_1_). *dac>dNek2* strongly inhibited retinal differentiation (H) and induced the accumulation of Fz3-RFP signals in the lateral marginal regions and an expansion of Fz3-RFP to the medial regions of the eye disc (white arrows) (H_1_). Anterior to the left. Scale bars, 50 μm. Relative differentiated regions were quantified and are presented in a dot plot. Each dot represents an individual measurement and horizontal bars the mean values (I) (n = 16–20, ^∗∗∗^p < 0.0001). The intensities of Fz3-RFP signals were quantified and the mean values are shown in a bar graph (J). The error bars indicate SEM (n = 6–10, ^∗∗∗^p < 0.0001).

We next addressed whether APC/C-dependent degradation of dNek2 in the G1-arrested region is required for the uniform progression of the MF. We induced *cdc16RNAi* specifically in the G1-arrested region by using *dac*-Gal4 as well as *hairy*-Gal4, whose activity is even more local and restricted to only a couple of rows of cells anterior to the MF within the G1-arrested region (Brown et al., 1995) (Figures 6A and 6E). *dac*>*cdc16RNAi1* caused a mild but significant reduction of the differentiated area in the eye disc (Figures 6A, 6B, and 6D), which was rescued by *dNek2RNAi1* co-induction (Figures 6C and 6D). *hairy*>*cdc16RNAi1* did not show any obvious effect on MF progression (data not shown). However, when co-expressed with Dcr-2 to enhance the RNAi efficiency, *hairy>cdc16RNAi1* also caused a clear delay in MF progression in the lateral regions of the eye disc (Figure 6F, white circle). These results suggest that the local APC/C activity in the G1-arrested region is critical for the coordinated progression of the MF.

We further asked whether dNek2 accumulation in the G1-arrested region is sufficient to trigger hyperactivation of Wg signaling. We found that *dac*>*dNek2* causes robust accumulation of Fz3-RFP reporter signals (Figures 6G, 6H, and 6J) and their massive expansion from the lateral margins toward the medial region of the eye discs (Figure 6H_1_, white arrows), concomitantly with significant inhibition of retinal differentiation and overgrowth of the anterior region (Figures 6G–6I). Collectively, our results suggest that APC/C^Fzr^ promotes uniform MF progression by suppressing Wg signaling via degradation of its positive modulator dNek2 upon synchronous G1 arrest ahead of the MF.

### The APC/C Is Stabilized in the MF Region in a Dpp Signaling-Dependent Manner

**Figure 7:**
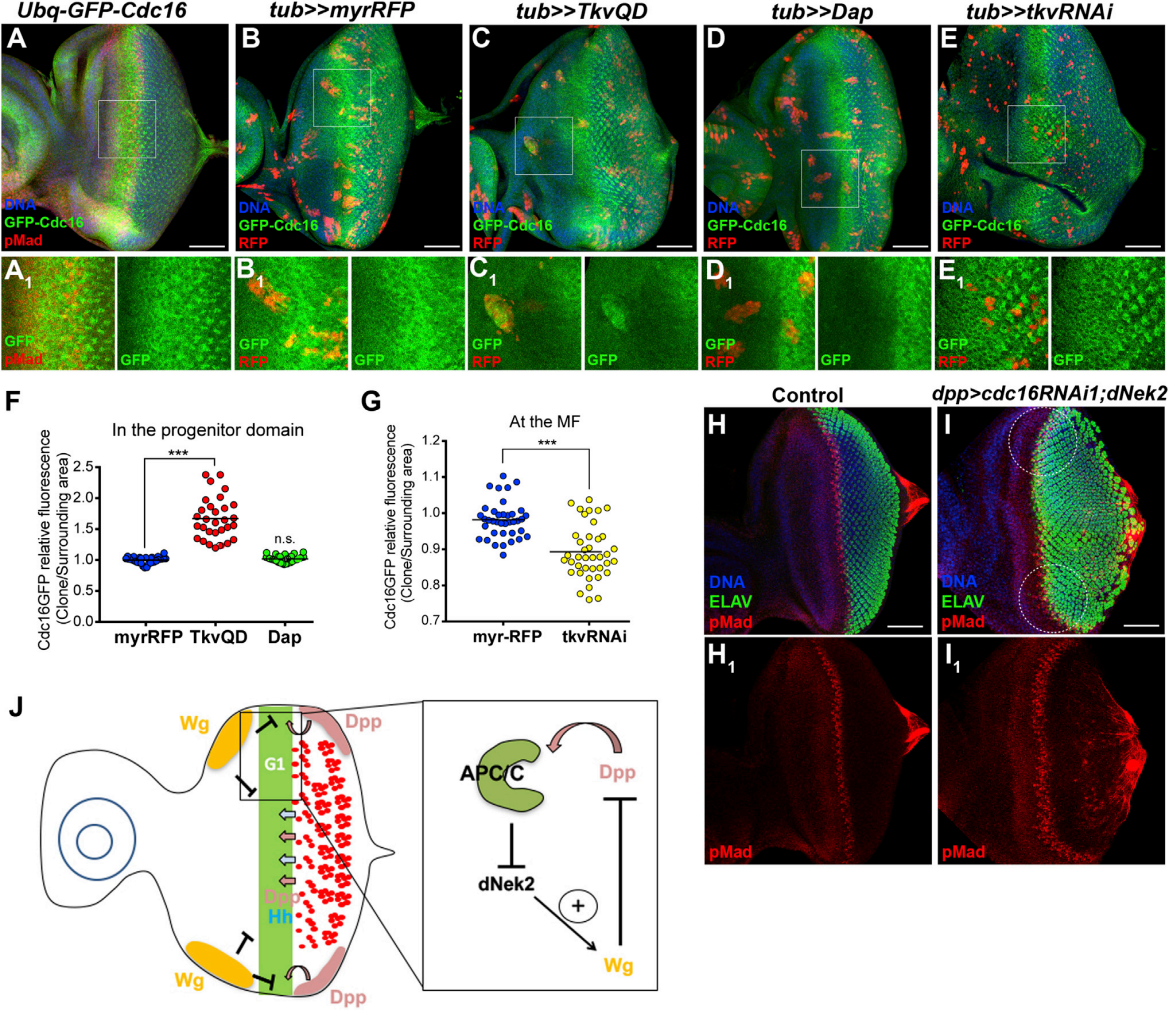
Cdc16 Is Stabilized in the G1-Arrested Region in a Dpp-Dependent Manner (A) Eye discs carrying the *pUbq-GFP-cdc16* transgene showed GFP-Cdc16 (green) (A_1_) accumulated at the G1-arrested region indicated by the co-localization with pMad (red) (A_1_). DNA in blue. Anterior to the left. Scale bar: 50 μm. (B–E) Eye imaginal discs carrying the *pUbq-GFP-cdc16* transgene, where clones expressing either control myrRFP (B, B_1_), TkvQD (C, C_1_), the CDK inhibitor, Dacapo (D, D_1_), or *tkvRNAi* (E, E_1_) were generated by Flp-out (identified by the expression of RFP, red). DNA in blue. Anterior to the left. Scale bar: 50 μm. Magnified images of the regions highlighted in B–E (white squares) are also shown (B_1_–E_1_). myrRFP expression did not affect the GFP-Cdc16 accumulation (B, B_1_), and TkvQD expression caused the accumulation of GFP-Cdc16 (green) in the clones generated anterior to the MF (C, C_1_), while expression of Dacapo did not change Cdc16 levels (D, D_1_). *tkvRNAi* clones partially inhibit the GFP-Cdc16 stabilization at the MF (E, E_1_). (F and G) Quantifications of GFP-Cdc16 levels in the Flp-out clones ectopically expressing the indicated genes, generated anteriorly to the MF (F) or at the MF (G). The fluorescence intensity of each clone was compared with the adjacent area and the ratios are represented in a scattered dot plot. Dots represent individual measurements and horizontal bars the mean values (n = 30–35, ^∗∗∗^p < 0.0001, n.s., no statistical difference). (H and I) Eye imaginal discs of control (H, *dpp*>GFP) and *dpp>cdc16RNAi1*;*dNek2* (I), stained for pMad (red) and ELAV (green). *dpp>cdc16RNAi1*;*dNek2* caused a delay in MF progression in the lateral margins (I) (white circles), without affecting Dpp activity (pMad, red) (H_1_, I_1_). Anterior to the left. Scale bars, 50 μm. (J) A schematic diagram of the model for the role of the APC/C in the uniform progression of the MF in the eye imaginal disc. Wg signaling (yellow) originating from the anterior lateral margins inhibits the progression of the MF and differentiation of photoreceptor neurons (red) by counteracting Dpp signaling (pink). In the G1-arrested region (green) ahead of the MF, Dpp signaling activity stabilizes the APC/C, promoting destruction of dNek2, a positive Wg modulator. Consequently, Wg signaling activity is suppressed, which facilitates the uniform progression of the MF across the eye disc.

Finally, we addressed how this non-cell-cycle APC/C function is regulated during eye development. It is known that the expression of Fzr is transcriptionally controlled during embryogenesis and eye imaginal disc development (Pimentel and Venkatesh, 2005, Sigrist and Lehner, 1997), pointing to a developmental control of APC/C activity. We analyzed the expression patterns of Cdc27 and Cdc16 in the eye disc using *pUbq-GFP-cdc27* and *pUbq-GFP-cdc16* transgenes (Huang and Raff, 2002). We found that, while GFP-Cdc27 is uniformly expressed throughout the eye-antenna imaginal disc (data not shown), GFP-Cdc16 exhibits a highly patterned expression in the eye disc: GFP-Cdc16 signals are relatively low in the anterior region but are highly accumulated in the MF region, coinciding with pMad signals (Figure 7A). In the differentiated region posterior to the MF, GFP-Cdc16 signals are exclusively concentrated in photoreceptors (Figure 7A). Dpp signaling is highly active in the MF to induce synchronous G1 arrest (Horsfield et al., 1998, Figures 2A and S3A–S3D). We therefore hypothesized that the MF region-specific Cdc16 accumulation may depend upon Dpp signaling. We generated clones that ectopically activate Dpp signaling by the expression of the constitutive active form of the Dpp type I receptor Thickveins (TkvQD) (Nellen et al., 1996). We observed a clear accumulation of GFP-Cdc16 in the TkvQD-expressing clones that were generated in the anterior progenitor region of the eye disc (Figures 7B, 7C, and 7F). To determine whether the GFP-Cdc16 accumulation is induced by G1 arrest independently of Dpp activity, we also ectopically expressed the Cdk inhibitor Dacapo to induce G1 arrest (Lane et al., 1996) and found no effect on GFP-Cdc16 levels (Figures 7D and 7F). Conversely, when we generated clones in which Dpp signaling was inactivated by *tkvRNAi* induction, we detected a small but significant reduction in GFP-Cdc16 levels in the clones at the MF (Figures 7E and 7G), pointing to the posttranslational control of Cdc16 levels by Dpp signaling. Finally, to examine the role of APC/C-dependent dNek2 degradation downstream of Dpp signaling, we induced *cdc16RNAi* together with the expression of untagged dNek2 in the Dpp active domain using a *dpp*-Gal4 driver. We observed a clear delay in MF progression in the lateral disc margins (Figures 7H and 7I, white circles), without any clear effect on Dpp signaling activity (Figures 7H_1_ and 7I_1_). Collectively, these results point to a critical role for the APC/C-Nek2 axis downstream of Dpp signaling in the regulation of the coordinated progression of retinal differentiation (Figure 7J).

## Discussion

In this study, we have uncovered a non-cell-cycle role for the master cell-cycle regulator APC/C in *Drosophila*. After the formation of the MF at the posterior tip of the eye disc, the APC/C downregulates Wg signaling in the G1-arrested region ahead of the MF through degradation of dNek2 (Figure 7J). This suppression of Wg activity is critical for the coordinated progression of retinal differentiation. If it is abrogated, progenitor cells are unable to differentiate or may take on different cell fates. Thus, the APC/C not only controls the cell cycle, but also directly regulates cell fate through the modulation of a major developmental signaling pathway, Wg/Wnt, during *Drosophila* eye development, establishing crucial molecular crosstalk between the master cell-cycle regulator and the major developmental pathway.

It has been known that the APC/C also plays a crucial role in postmitotic processes (Choi et al., 2014, Yang et al., 2010). However, due to the indispensability of its function for cell viability, it has been challenging to scrutinize, specifically, cell-cycle-independent functions of the APC/C. By taking advantage of the hypomorphic nature of in vivo RNAi and knocking down the individual components of the multisubunit APC/C complex, we were able to observe a distinct eye phenotype: a severe differentiation defect in the adult eye with an indication of cell-fate conversion (Figure 1). We have concluded that this phenotype is not due to cell-cycle defects for the following reasons. First, unlike the previously reported *fzr* and *shtd* mutants (Pimentel and Venkatesh, 2005, Tanaka-Matakatsu et al., 2007), the synchronized G1 arrest is maintained in the eye disc (Figures 1C and 1D). Second, these eye discs grow to a normal size (Figure 1G) and the mitotic index increase does not correlate with the degree of differentiation inhibition (Figures 1F and S2F). Third, the Cdc16 knockdown, specifically in the MF region where the majority of cells are arrested in G1 phase, also inhibits retinal differentiation (Figures 6B and 6F). Finally, dNek2 depletion rescues the differentiation defect, but not the mitotic index increase, of *ey>cdc16RNAi1* (Figure 4).

The differences in the phenotypes observed upon RNAi between the APC/C subunits may reflect different molecular functions of individual components within the APC/C. According to the subatomic model of the human APC/C structure (Chang et al., 2015), Cdc16, Apc1, and Apc5, which showed the differentiation failure phenotype upon *ey*-Gal4-driven RNAi (Figure S1, Table S1), interact directly to form a part of the scaffold on the opposite side to the catalytic core. We showed previously that human Nek2A is recognized by the APC/C through direct interaction with APC/C core subunits (Hayes et al., 2006). Thus, partial depletion of these subunits might generate a defective APC/C complex that specifically lacks the ability to recognize dNek2 while maintaining ubiquitination activity toward canonical substrates such as cyclin B, enabling cell-cycle progression. Alternatively, dNek2 might be a less processive substrate and more sensitive to a decrease in the overall APC/C activity than other APC/C substrates (Williamson et al., 2011). We also cannot rule out the possibility that those three subunits may regulate dNek2 levels independently of the APC/C by either forming a subcomplex or as monomers. More detailed analyses are required to determine the cause of the phenotypic difference between APC/C components.

We have concluded that APC/C inactivation inhibits retinal differentiation through ectopic activation of Wg signaling. In addition to the phenotypic similarity to the effects of ectopic Wg activation in the eye disc (Figure 1) (Baonza and Freeman, 2002), we also observed robust induction of the Fz3-RFP reporter (Figure 2). Our results strongly suggest that the APC/C downregulates Wg signaling specifically in the G1-arrested region ahead of the MF (Figures 5 and 6). This local Wg downregulation is critical for MF progression, particularly in the lateral marginal areas where cells are exposed to a higher concentration of Wg ligands due to their closer proximity to the Wg-producing cells (Treisman and Rubin, 1995). Thus, by suppressing Wg signaling ahead of the MF, the APC/C may negate the local difference in Wg signaling to ensure the uniform movement of the MF across the dorsoventral axis.

We have identified dNek2 as the mediator between APC/C activity and Wg signaling (Figures 3 and 6). dNek2 recently emerged as a positive Wg modulator that directly binds and phosphorylates Dsh (Schertel et al., 2013); however, its role in Wg signaling regulation in the physiological condition remained unclear. Our study is the first demonstration of the in vivo role of dNek2 in Wg signaling regulation. dNek2 is degraded by the APC/C^Fzr^ specifically in the G1-arrested region ahead of the MF (Figure 5), which allows local suppression of Wg signaling to move the MF forward, while maintaining the Wg activity in the anterior progenitor domain to promote cell proliferation and prevent ectopic MF formation. Thus, dNek2 may act as a cell-cycle-dependent Wg enhancer that, through its cell-cycle-dependent oscillation, alters the responsiveness of the recipient cells to Wg signaling according to their cell-cycle state. Accumulating evidence suggests the cell-cycle-dependent regulation of Wnt signaling (Davidson and Niehrs, 2010). A role of dNek2 in such cell-cycle control of Wnt signaling remains to be elucidated.

Is this Nek2 function in Wnt signaling regulation evolutionarily conserved? It was shown that human Nek2 also phosphorylates Dsh and, when co-expressed with CK1ɛ, synergistically promotes the Wnt signaling activation induced by Dsh expression (Cervenka et al., 2016). Importantly, human Nek2 has been established as a key centrosome regulator (Fry, 2002). It was shown that the Wnt components, Dsh and β-catenin, are also localized at the centrosome and function downstream of Nek2 to regulate centrosome separation (Mbom et al., 2013). It is important to determine how the centrosomal function of Nek2 and the Wnt pathway components is linked to the canonical Wnt signaling.

Our results point to a role of the Dpp signaling of the transforming growth factor β (TGF-β) family in the activation of the APC/C (Figure 7). Interestingly, this regulation appears to be mediated by the stabilization of Cdc16, which, together with our main finding, highlights the importance of posttranslational regulations in developmental signal transduction. In mammals, APC/C^CDH1^ also directly regulates the activation of TGF-β signaling by degrading the transcriptional co-repressor SnoN upon TGF-β stimulation (Stroschein et al., 2001, Wan et al., 2001). As the D box motif responsible for the SnoN degradation is conserved in the *Drosophila* SnoN ortholog, Dpp may form a positive feedback loop for robust activation through APC/C-mediated proteolysis by auto-activation through SnoN degradation and suppression of the antagonistic Wg activity (Figure 7J). A potential role for the APC/C in this crosstalk between the two major signaling pathways will be a subject of future studies.

APC/C^Fzr/CDH1^ is required for the induction and maintenance of G1 arrest for terminal differentiation and cellular quiescence (Eguren et al., 2011, Yang et al., 2010). We have demonstrated that APC/C^Fzr^ regulates the activity of the canonical Wnt signaling pathway upon synchronized G1 arrest through dNek2 degradation to initiate photoreceptor differentiation. The accompanying study from the Mlodzik group has also reported that the APC/C^Fzr^-dependent dNek2 degradation in postmitotic cells is also required for the establishment of planar cell polarity through the regulation of the non-canonical Wnt signaling pathway in the *Drosophila* eye and wing (Weber and Mlodzik, 2017). It is therefore tempting to speculate that the APC/C^Fzr^ may ensure proper differentiation and patterning by coordinating G1 arrest and developmental signaling. Such coordination between the cell cycle and developmental signaling may be crucial for the development of multicellular organisms that requires strict coordination between cell proliferation and differentiation.

## Experimental Procedures

### Analysis of Adult Eye Phenotypes Induced by RNAi against APC/C Subunits

RNAi was induced by crossing the RNAi lines with either the *ey*-Gal4 or GMR-Gal4 driver line. The compound eyes of at least 50 adult flies were examined for each sample under a stereomicroscope (Leica S8 APO) equipped with a digital camera (TrueChrome HDMI Camera, GT Vision), and several representative pictures for each line were taken if any alterations on the eye sizes or structures were detected.

The eye phenotypes with *ey*-Gal4 were qualitatively classified into the following five categories, according to the effects on the eye sizes, shapes, and structures: class 0, “no effect,” the eye shows no detectable alterations from the wild-type; class 1, “small reduction,” the eye size was reduced down to two-thirds of the normal size without obvious effect on the pattern; class 2, “medium reduction,” the size was reduced to two-thirds to one-third of the normal size with normal polarity; class 3, “strong reduction,” the eye was tiny or completely absent, associated with high lethality; class 4, “eye-shape defect with cell-fate loss,” these flies present a protruding eye and frequently form ectopic structures within the adult retina, in concurrence with the eye size reduction down to 50% of the normal size.

The eye phenotypes with the GMR-Gal4 driver were qualitatively classified into the following four categories: class 0, “no phenotype,” the eye shows no detectable alterations from the wild-type; class 1, “missing bristle,” an eye missing the eye bristles but otherwise wild-type; class 2, “slight rough,” an eye with noticeable roughness in some patches; class 3, “strong rough eye,” an eye showing strong roughness in the entire eye with occasional size reduction.

For the quantification of the ectopic structures in the adult retina, six independent vials were analyzed where at least 50 F1 adult flies were scored by visual inspection for: normal eye, rough eye, ectopic head structure in the retina field, and ectopic antenna-like structure in the retina field.

### Measurement of Area Sizes, Mitotic Indices, and Fluorescent Signal Intensities in the Eye Imaginal Discs

The area sizes of projected images were measured using the Polygon selection tool of ImageJ 1.50i (NIH). The eye disc areas were determined by measuring the eye fields in the eye-antenna imaginal discs and represented as the percent of control. The differentiated areas were determined by measuring the size of the ELAV-positive area size and represented as a ratio of the eye disc area.

For the signal intensity measurements, projected images were quantified in the selected areas using the Polygon selection tool of ImageJ 1.50i. The fluorescence intensities of dNek2-GFP and pMad signals were measured in the entire eye field of the imaginal disc and represented as the mean value of fluorescence intensity.

The signal intensity values of dNek2-GFP levels at the MF on the *dac-*Gal4-driven conditions were measured using the plot profile tool on the highlighted area (256 × 256 px) and the profile represented as the percentage of the maximum signal. For each signal profile, a minimum of three different eye discs were used and the graphical output was performed with GraphPad Prism 6.0.

For the measurement of the Fz3-RFP and DPP-lacZ signal intensities, the proximal region of the antenna disc was used as the background signal to define the signal-positive areas in the eye filed. The signal intensities were measured in the signal-positive areas.

The mitotic index was automatically determined using the Analyze Particles tool of ImageJ 1.50i in projected images with PH3 as a mitotic marker and presented as the number of mitotic cells per eye imaginal disc.

For the quantification of the effect of Dpp modulation on Cdc16-GFP stabilization, the Flp-out clones were marked with RFP and the area was delimited using the Polygon selection tool of ImageJ. The same area was considered to determine the GFP levels and compared with the adjacent area. The values are represented as ratio clone/adjacent area and dots represent individual clones and horizontal bars show the mean values.

GraphPad Prism 6.0 was used for statistical analysis and generation of the graphical output.

### In Vitro APC/C-Dependent Destruction Assay

Destruction assays were performed as described previously (Hayes et al., 2006). The details of the preparation of recombinant Mes1 and *Drosophila* Fzr proteins can be found in the Supplemental Experimental Procedures.

### Statistical Analyses

Statistical analysis was performed with GraphPad Prism 6.0. The D'Agostino-Pearson omnibus normality test was applied to datasets to assess data distribution. For normally distributed data, an unpaired t test was used. For non-normally distributed data, the Mann-Whitney U test was used. Differences are considered significant with a p value less than 0.05. ^∗^0.01 < p ≤ 0.05, ^∗∗∗^p < 0.0001.

## Author Contributions

T.M. designed and conducted most of the experiments and contributed to writing the manuscript. F.M. designed and conducted the in vitro destruction assays and cell culture work and helped with preparation of the reagents and the figures. F.F. contributed to preparing the reagents and initial characterization of the *dNek2-GFP* transgenes. Y.K. supervised the project, wrote the manuscript, and obtained the funding.

## Supplemental Information

Document S1. Supplemental Experimental Procedures, Figures S1–S6, and Table S1

Movie S1. Z-Section Animation to Show the Ordered Epithelial Structure of the Control Eye Imaginal Disc, Related to Figure 1

Movie S2. Z-Section Animation to Show the Epithelial Structure of the ey>cdc16RNAi1 Eye Imaginal Disc, Related to Figure 1

Movie S3. Z-Section Animation to Show the Epithelial Structure of the ey>cdc16RNAi1:P35 Eye Imaginal Disc, Related to Figure 1

Document S2. Article plus Supplemental Information
