# Supplementary material for: The APC/C Coordinates Retinal Differentiation with G1 Arrest through the Nek2-Dependent Modulation of Wingless Signaling
Source: Dev Cell. 2017 Jan 9;40(1):67–80. doi: 10.1016/j.devcel.2016.12.005 (PMC5225405; doi:10.1016/j.devcel.2016.12.005)
Supplement: Document S1. Supplemental Experimental Procedures, Figures S1–S6, and Table S1 [file mmc1.pdf]

Developmental Cell, Volume *40*

Supplemental Information

The APC/C Coordinates Retinal Differentiation with G1 Arrest through the Nek2-Dependent Modulation of Wingless Signaling

Torcato Martins, Francesco Meghini, Francesca Florio, and Yuu Kimata

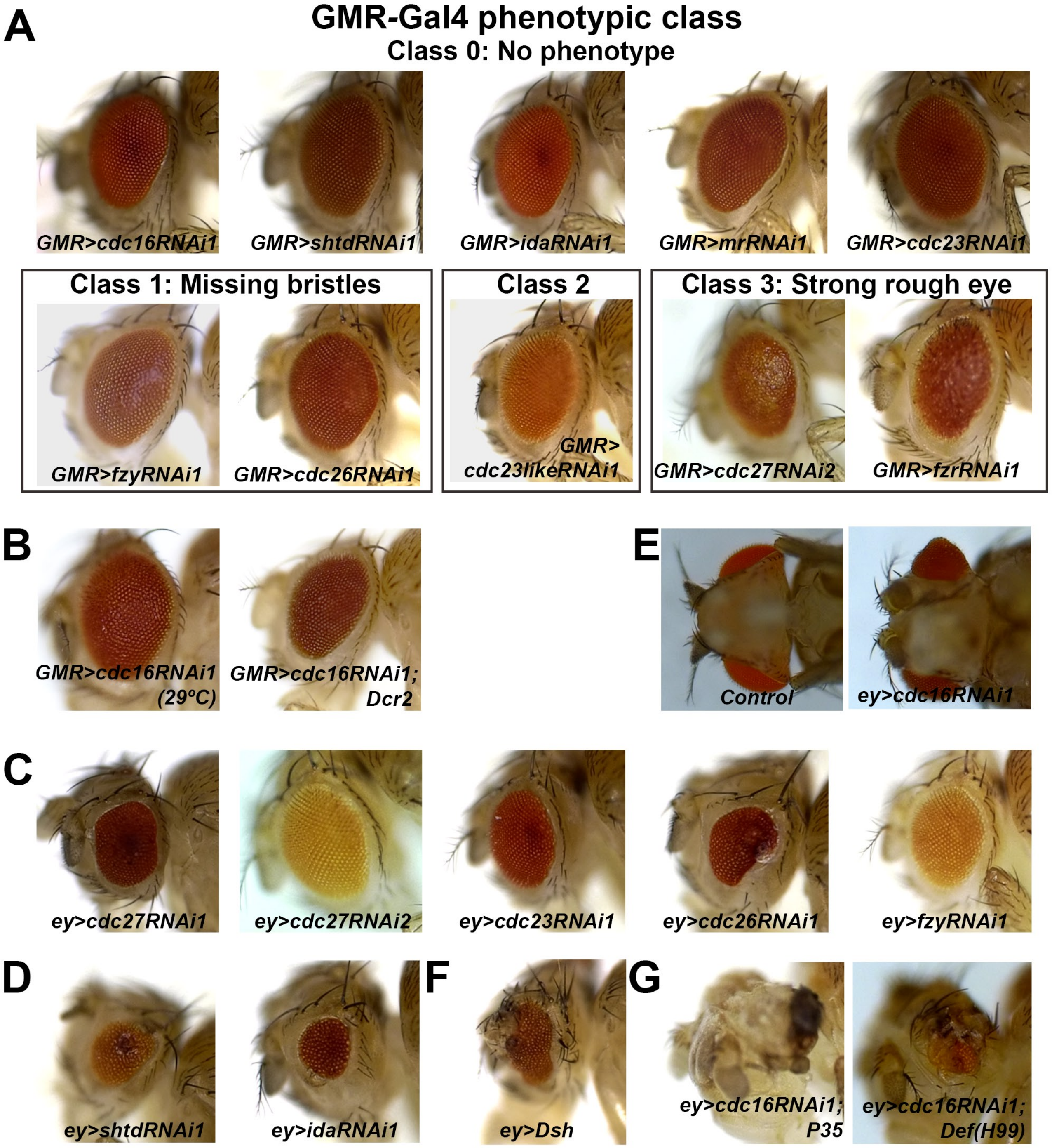

Figure S1, related to Figure 1. Adult eye phenotypes caused by RNAi against APC/C subunits using the ey-Gal4 or GMR-Gal4 drivers.

A) The adult eye phenotypes observed upon GMR-Gal4-driven RNAi of APC/C components were classified in four classes: Class 0 – No effect on the retina; Class 1 –smooth and patterned eyes but the eye bristles are missing; Class 2 – noticeable roughness in some patches on the eye; Class 3 –strong roughness in the whole eye with occasionally reduction in eye size.

B) (Left) the *GMR>cdc16RNAi1* was induced at an higher temperature to increase the Gal4 efficiency. (Right) *GMR>cdc16RNAi1* was co-induced with Dcr2 to improve the RNAi efficiency.

C) The adult eye phenotypes observed upon ey-Gal4-driven RNAi of APC/C components.

D) The adult eye phenotypes observed upon RNAi of Shtd or Ida.

E) The head ventral views of the control and cdc16RNAi1. The protrusion of the eye structures was evident in the *ey>cdc16RNAi1* adult heads.

F) The adult eye phenotypes observed upon ectopic induction of the Wg component, Dsh. As previously described, Wg ectopic activation results in ectopic head/antenna structures in the retina field.

G) The adult eye phenotypes caused by *ey>cdc16RNAi1* with the co-expression of P35 or in the Def(3L)H99 background.

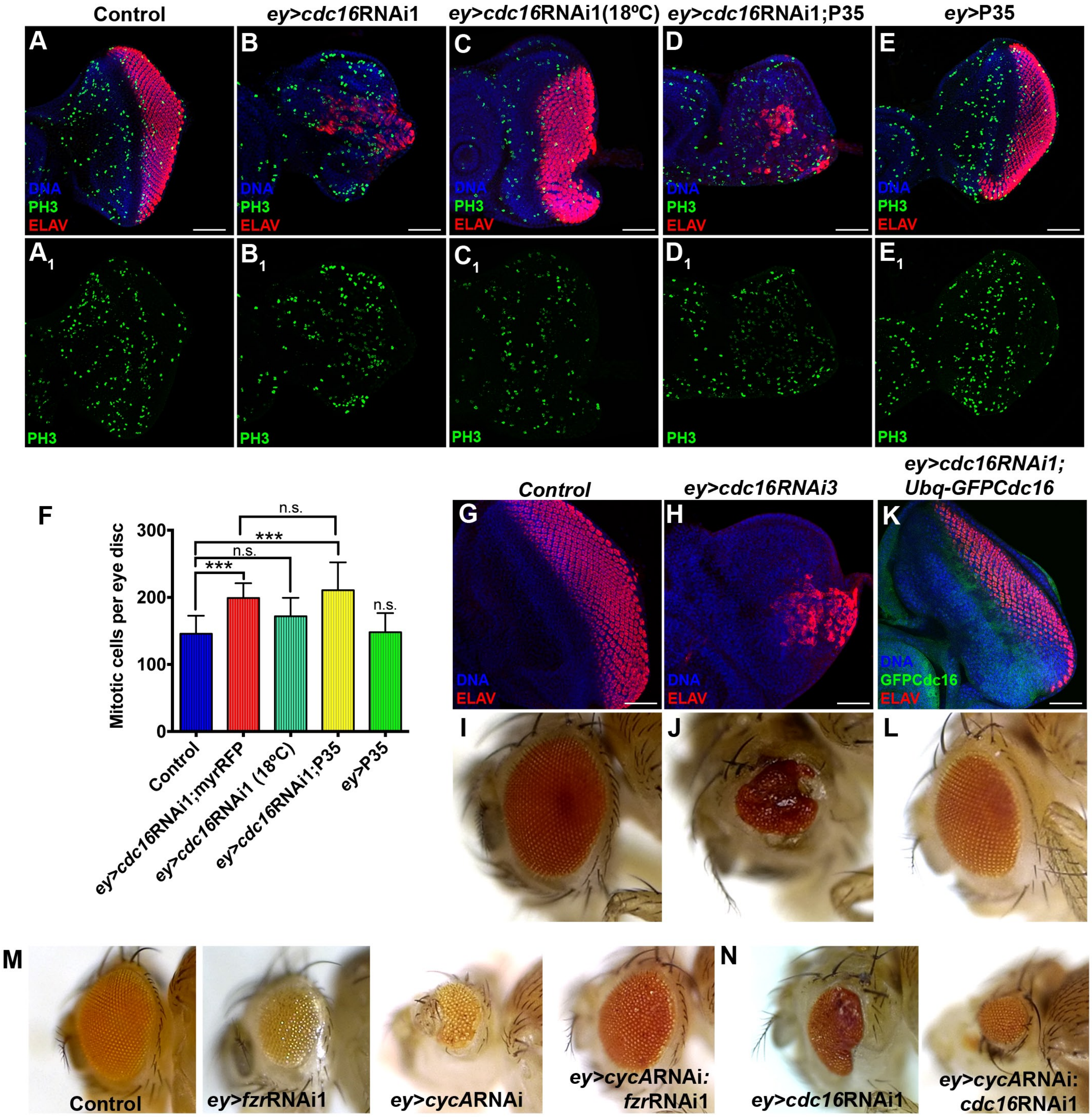

Figure S2, related to Figure 1. Cdc16 depletion causes a mild increase in mitotic index, which is not enhanced by apoptosis inhibition. Validation of the specificity of the cdc16 RNAi lines and genetic interactions with Cyclin A.

A-E1) Larval eye imaginal discs of the indicated genotypes stained for DNA (blue), PH3 (green) and ELAV (red). Depleting Cdc16 (**B-B1**) causes mild but significant mitotic arrest when compared to control (**A-A1**). Reduced cdc16RNAi1 efficiency by temperature shift had no impact on the mitotic cell number (**C1**, at 18ºC) while still disrupting the uniform MF progression **(C)**. Eye discs co-expressing P35 and cdc16RNAi (**D-D1**) have a strong impact in photoreceptor differentiation (**D**) with no significant differences in the number of mitotic cells are seen comparing to cdc16RNAi1 (**D1**). Overexpression of P35 alone showed not impact on photoreceptor differentiation (**E**) nor in the number of mitotic cells (**E1**). **F)** Graphic representation of an automatic quantification of the number of mitotic cells per eye disc of the indicated genotypes. Data are represented as the mean ± sd (n=10-15, ***p<0.0001 and n.s. means no statistical difference). **G-K**) Representative images of the eye imaginal discs stained for DNA (blue) and the neuronal marker, ELAV (red). Cdc16 depletion using a different RNAi line (**H**) strongly suppressed neuronal differentiation when compared to the control (**G**). The pUbq-GFP-Cdc16 transgene restored the neuronal differentiation induced by cdc16 RNAi1 (**K**). **I-L**) Adult eyes of the genotypes analysed in G-K. Cdc16 depletion using a different cdc16 RNAi line 3 (KK103583) caused the eye-shape defect phenotype in the adult eyes (**J**). The pUbq-GFP-cdc16 transgene rescued the phenotype induced by cdc16 RNAi1 **(L)** and the adult eyes were similar to the control (**I**). **M, N)** Representative images of the adult retinas of the control, fzrRNAi, cycARNAi, cycARNAi;fzrRNAi (**M**), cdc16RNAi and cycARNAi;cdc16RNAi (**N**). cycA RNAi caused strong reduction in adult eye size (**M**). fzr RNAi and cycA RNAi partially suppressed the adult eye phenotypes of each other (**M**). cdc16 RNAi did not affect the cycA RNAi phenotype (**N**). Scale bars: 50 µm.

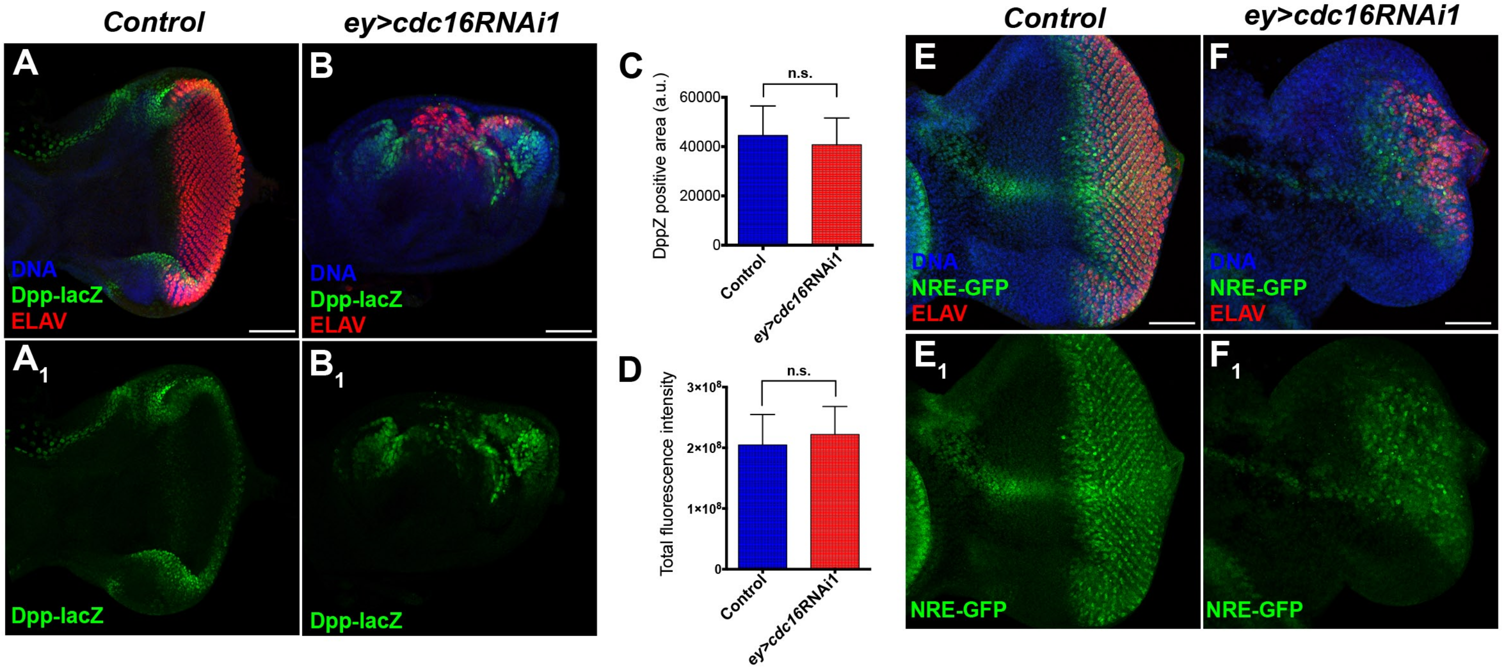

Figure S3, related to Figure 2. APC/C knockdown has no effect on Dpp expression and Notch activity
A-B1) Representative images of the eye imaginal discs of: eyG4 (Control, **A**) and *ey>cdc16RNAi1* (**B**) reporting dpp expression (Dpp-lacZ, green). In the control discs, Dpp-lacZ signals were detected at the Morphogenetic furrow and the lateral eye disc margins (**A1**). Dpp-lacZ enhanced the differentiation defect caused by cdc16RNAi (**B**). Nonetheless, the expression levels of Dpp-lacZ were not significantly affected by cdc16RNAi (**B).** **C-D**) The Dpp-lacZ positive areas (**C**) and total intensities of the Dpp-lacZ signals (**D**) of the control and *ey>cdc16RNAi1* were measured in the individual eye imaginal discs. Data are represented as mean ± SEM (n=10, n.s. means no statistical difference). **E, F1**) Representative images of the eye imaginal discs of ey-Gal4 (Control, **E**) and *ey>cdc16RNAi1* (**F**) carrying the Notch signalling activity reporter, NRE-GFP (green). The eye discs were stained with DAPI (blue) and the differentiation marker, anti-Elav (red). In the control, NRE-GFP was induced in the medial region and in the Elav positive posterior region of the eye discs (**E, E1**). cdc16 RNAi did not appear to affect the expression patterns or the levels of the NRE-GFP in the eye imaginal discs (**F, F1).** Scale bars: 50 µm.

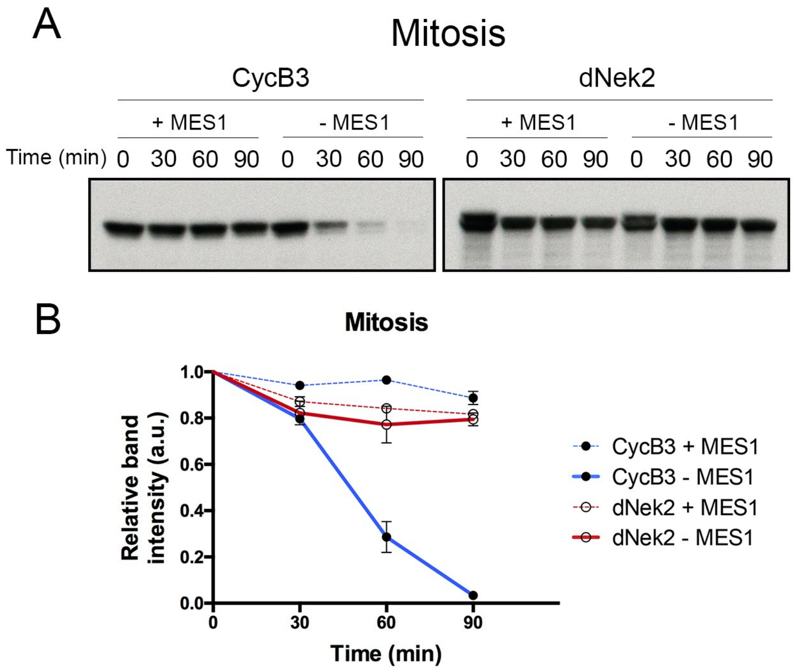

Figure S4, related to Figure 3. dNek2 is not targeted by APC/C^{Fzy} *in vitro*.

A) *In vitro* destruction assays of CycB3 and dNek2 in mitotic egg extracts in the presence or absence of an APC/C inhibitor, Mes1. In the absence of Mes1, APC/C efficiently degraded CycB3 within 90min (left panel, -Mes1) but not dNek2 (right panel, -Mes1).

B) Quantification of the relative CycB3 or dNek2 band intensities (relative to time 0) in the mitotic destruction assays (n=3, error bars represent SEM).

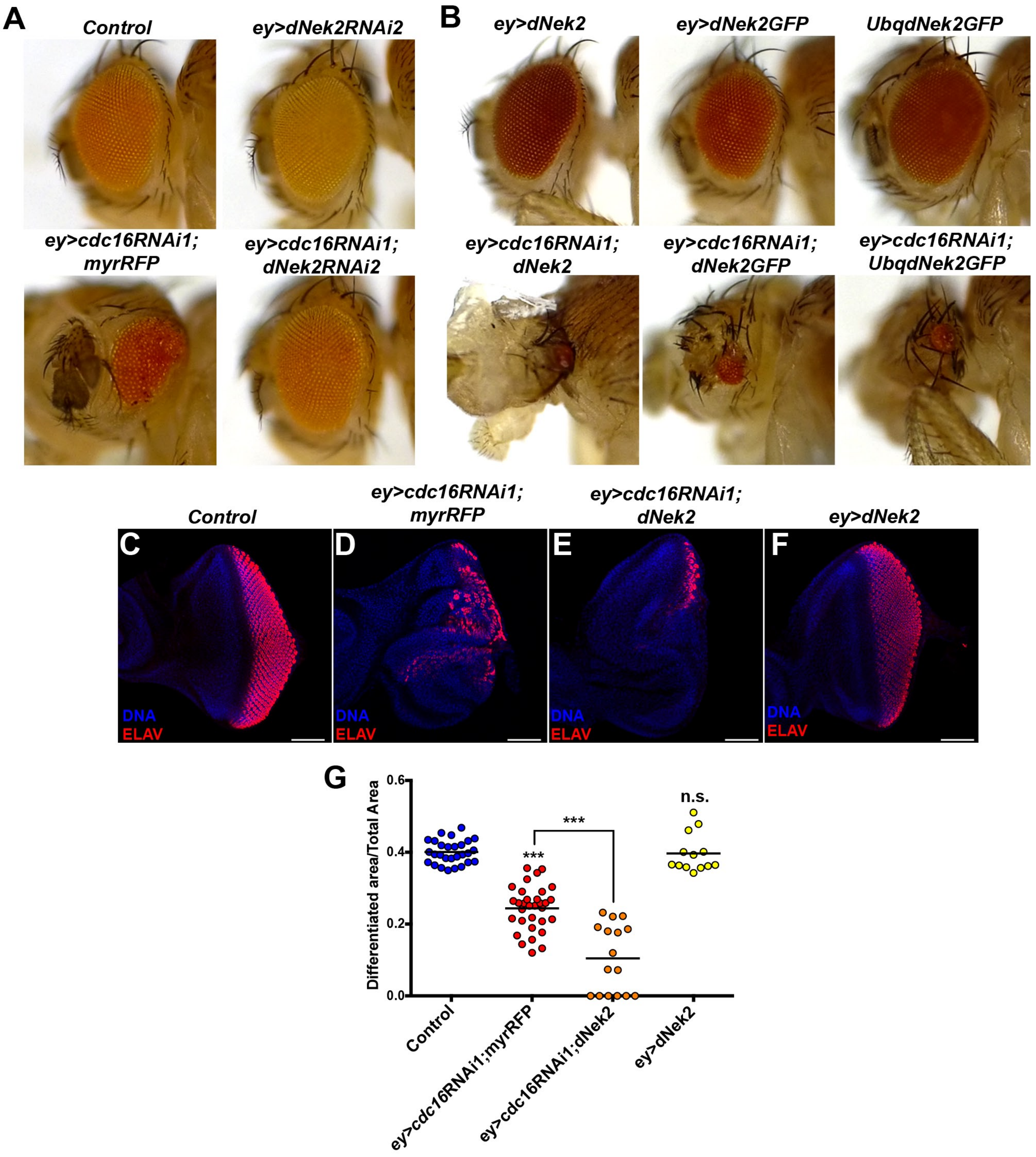

Figure S5, related to Figure 4. Overexpression of dNek2 enhances the differentiation impairment caused by Cdc16 depletion
A) Adult eyes of control (top left) and *cdc16RNAi* depleted eye imaginal discs (bottom left). dNek2 depletion using a different RNAi line (dNek2RNAi2) did not affect the adult eyes (top right), whilst it fully rescued the eye-shape defect phenotype caused by Cdc16 depletion in the adult eyes (compare the bottom left and the bottom right panels). UAS-myr-RFP was used as the negative control and as reference for the rescue of the *cdc16RNAi* phenotype. **B**) Representative images of the adult eyes of the indicated genotypes. Neither ey-Gal4-induced overexpression of untagged dNek2 (top left) or dNek2-GFP (top middle), nor the Ubq-dNek2-GFP transgene (top right) affect the adult eye morphology. All the dNek2 overexpression conditions significantly enhanced the differentiation impairment caused by *cdc16RNAi* (bottom panels). **C-F**) Representative images of the eye imaginal discs stained for DNA (DAPI, blue) and the neuronal differentiation (ELAV, red). The expression of untagged dNek2 alone did not have any detectable effect on the eye imaginal discs (**F**) resembling control eye imaginal discs (**C**). In the absence of Cdc16, dNek2 significantly enhanced (**E**) the differentiation impairment caused by *cdc16RNAi* alone (**D**). **G**) Relative differentiated regions of the indicated genotypes were measured and represented as a disstribution. Dots represent individual measurements and horizontal bars show the mean values. (n=15-30, ***$p<0.0001$ and n.s. means no statistical difference).

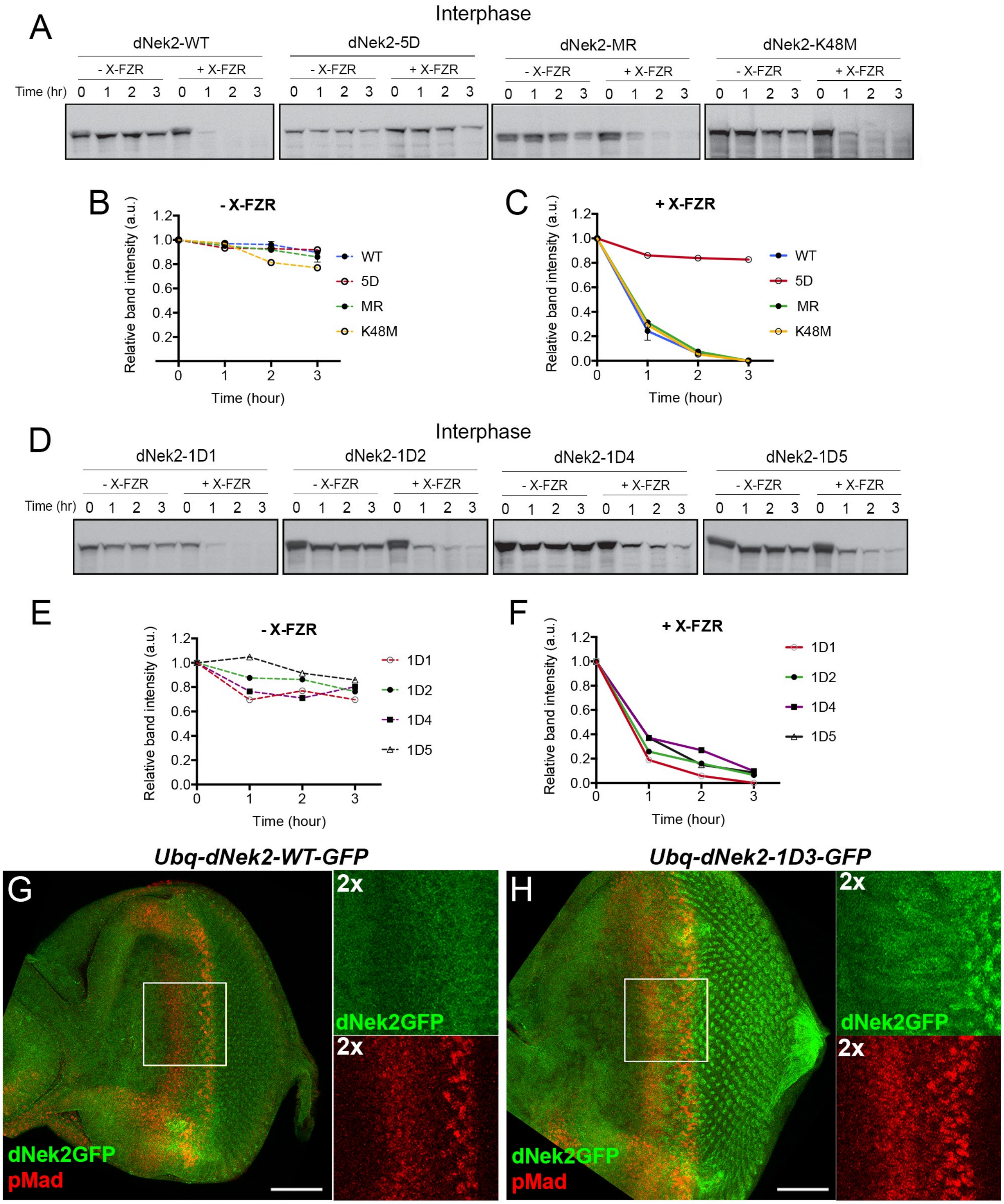

Figure S6, related to Figure 5. The D-box-like motif at position 355 is the main APC/C degron of dNek2.
A) *In vitro* destruction assays of dNek2-WT, dNek2-5D, dNek2-MR and dNek2-K48M in interphase egg extracts. The dNek2-5D mutation completely stabilised dNek2, whereas dNek2-MR or dNek2-K48M had no effect on dNek2 stability (+ X-FZR). (**B, C**) Quantification of the relative dNek2-WT/5D/MR/K48M band intensities (relative to time 0) in the absence (**B**) or presence (**C**) of X-Fzr in the interphase destruction assays (n=3, error bars represent SEM). **D**) The *in vitro* destruction assays in interphase egg extracts using the D-box mutant constructs of dNek2-1D1, dNek2-1D2, dNek2-1D4 and dNek2-1D5 as substrates. **E,F**) The band intensities of the various dNek2 mutants were quantified and the values in the absence of X-Fzr (**E**) or in the presence of X-Fzr (**F**) were plotted in the line graphs (n=1 for the single D-box-like motif mutants, n=2 for dNek2-1D3). **G,H**) Third-instar eye imaginal discs of the indicated genotypes stained for DAPI (blue) and pMad (red). dNek2-WT-GFP levels drop at the G1 arrest region (**G**, co-stained with pMad, 2x magnification right panels). Mutation in the 3rd D-Box of dNek2 is sufficient to stabilise dNek2GFP at the G1 arrest region (**H**, 2x magnification right panels).

Gene	RNAi stock		*ey*-Gal4	GMR-Gal4
APC1/Shattered	1	GD29072	4	0
	2	KK110343	0	0
APC2/Morula	1	KK106986	3	0
APC3/Cdc27	1	TRiP31716	2	2
	2	GD35986	1	3
APC4	1	GD38989	0	0
APC5/Ida	1	GD18845	4	0
	2	KK108634	3	0
APC6/Cdc16	1	GD44869	4	0
	2	GD52667	4	0
	3	KK103583	4	0
APC7	1	KK110729	0	0
APC8/Cdc23	1	KK107806	1	0
	2	TRiP31735	0	0
Cdc23-like (CG31687)	1	GD21393	0	2
APC10	1	KK107831	1	0
APC11/LemmingB	1	KK103649	0	0
Cdc26/CG17343	1	KK102248	2	1
APC15/CG15237	1	KK106899	1	0
	2	GD47254	0	0
Cdc20/Fzy	1	GD40500	2	1
	2	GD44834	1	1
	3	KK105114	1	1
Cdh1/Fzr	1	GD25550	3	3
	2	GD25553	2	3

ey-Gal4 phenotypic class:

0 - No phenotype
1 - Small reduction
2 - Medium reduction
3 - Strong reduction
4 - Eye shape defect with cell fate loss

GMR-Gal4 phenotypic class:

0 - No phenotype
1 - Missing bristles
2 - Slight rough eve
3 - Strong rough eye

Table S1, related to Figure 1. The summary of the adult eye phenotypes observed upon RNAi against APC/C components using ey-Gal4 or GMR-Gal4.

The adult eye phenotypes induced by RNAi against the indicated APC/C subunits or the APC/C activators using ey-Gal4 were classified into the following five classes according to the effects on the retina size, the eye structure: Class 0 – No effect on the retina formation; Class 1 – Small reduction in retina size; Class 2 – Significant reduction in retina size; Class 3 –very strong reduction in eye size associated with high lethality; Class 4 – protruding eyes with mild size reduction and ectopic antenna-like structures. Representative pictures of each class and the more detailed description of the classification can be found in Figure 1 and the Experimental procedures, respectively.

The adult eye phenotypes induced by the GMR-Gal4 driver were classified into the following four classes according to the degree of the effects on the retina patterning and the bristle formation: Class 0 – No effect on the retina; Class 1 –smooth and patterned eyes but the eye bristles are missing; Class 2 – noticeable roughness in some patches on the eye; Class 3 –strong roughness in the whole eye (very irregular pattern) with occasionally reduction in eye size. Representative pictures of each class and the more detailed description of the classification can be found in Figure S1 and the Experimental procedures, respectively.

Supplemental Movies

Supplemental Movie 1, Related to Figure 1. – Z-sections animation to show the ordered epithelial structure of the Control eye imaginal disc. The eye imaginal disc on the left shows the CycB (green) staining to identify the G1 arrest region (absence of CycB signal) and ELAV for the differentiated photoreceptors (red). The right image shows the nuclei (DAPI, blue) organisation on the control epithelium. The Z-step interval is 2 µm.

Supplemental Movie 2, Related to Figure 1. - Z-sections animation to show the epithelial structure of the *ey>cdc16*RNAi1 eye imaginal disc. The eye imaginal disc was stained for CycB (green) to identify the G1 arrest region (absence of CycB signal) and for the differentiated photoreceptors (ELAV, red). The right image shows the nuclei (DAPI, blue) organisation on the *ey>cdc16*RNAi1 epithelium. The Z-step interval is 2 µm.

Supplemental Movie 3, Related to Figure 1. - Z-sections animation to show the epithelial structure of the *ey>cdc16*RNAi1:P35 eye imaginal disc stained for CycB (green) and ELAV (red). Inhibition of apoptosis in the absence of *cdc16*, disrupts the MF formation and the eye disc epithelium present several disorganised folds. The appearance of different folds is clearer on the nuclear organisation (DAPI, blue, right panel). The Z-step interval is 2 µm.

Supplemental Experimental Procedures

Antibodies

The following antibodies were obtained and used at the indicated dilution for immunofluorescence or western blotting: mouse anti-CycA (Developmental Studies Hybridoma Bank, DSHB, A12, 1:100), mouse anti-CycB (DSHB, 1:100), rat anti-ELAV (DSHB, 1:250), rabbit anti-PH3 (Upstate, 1:750), rabbit anti-β-Gal (Cappel, 1:2000), mouse anti-GFP (Sigma-Aldrich, 11814460001, 1:1.000), mouse anti-Dll (Duncan et al., 1998), rabbit anti-pMad (Phospho-Smad1/5 (Ser463/465), Cell Signaling, 1:1000), mouse anti-Cdc2 (PSTAIR, Sigma, 1:2000) and anti-α-tubulin (Sigma-Aldrich, T6199, 1:1000).

***Drosophila* strains and husbandry**

All crosses were raised at 25°C under standard conditions unless stated otherwise. The following *Drosophila* lines were used: *Oregon R* (as the wild type), *ey-Gal4, GMR-Gal4, Dpp-Gal4, dac-Gal4(Tavsanli et al., 2004)*, *pUbq-GFP-Cdc16* (Huang and Raff, 2002), UAS-TkvQD (Nellen et al., 1996), *Fz3-RFP* (Olson et al., 2011) and *NRE-GFP* (gift from Sarah Bray) and UAS-Dacapo (Lane et al., 1996). The following fly stocks were obtained from Bloomington *Drosophila* Stock Center (BDSC): *UAS-P35* (#5072), *Dpp-lacZ* (#8411), *UAS-myrRFP* (#7118), *UAS-dNek2RNAi2* (#28600), *UAS-Cdc27RNAi1* (#31716), Df(3L)H99 (#1576), *UAS-Dicer2* (#24650) and *hairy-Gal4 (#1734)*. The *UAS-dNek2* (F001561), *UAS-dNek2-3xHA* (F000751) and UAS-Dsh (F001519) lines were obtained from FlyORF (Bischof et al., 2013). The other RNAi lines used were obtained from the Vienna *Drosophila* RNAi Center (VDRC) and the reference can be found on the Table S1. Not listed in the Table S1: dNek2RNAi1 (#KK103408), TkvRNAi (#GD3059) and CycARNAi (#GD32421). pUbq-dNek2-GFP and UAS-dNek2-GFP lines were generated using the embryo injection service at the Fly Facility at Department of Genetics, University of Cambridge.

Immunostaining of *Drosophila* eye imaginal discs

Third instar imaginal discs were dissected in PBS and fixed in 3.7% formaldehyde for 30 minutes. Triple washed with PBS-Triton 0.1% and incubated with the primary antibodies diluted in PBST overnight at 4ºC. After another three washes in PBST, incubated with the appropriate Alexa-Fluor conjugated secondary antibodies (Molecular Probes) and DAPI for 2 hours at RT and after three washes the eye discs were mounted in 50% Glycerol. Images were taken using the Nikon C2 confocal microscope and processed using the NIS-Elements software or image J and were assembled with Adobe Photoshop.

Mitotic recombination

Mitotic recombination was induced using the Flp/FRT method (Xu and Rubin, 1993). TkvQD, TkvRNAi, myrRFP or Dacapo clones were induced by heat shock (1 hour at 37°C) at 72 hours after egg-laying and dissected and analysed 48h after the heat shock.

Plasmid DNA construction

The cDNA clone of d*Nek2* was obtained from Drosophila Genomics Resource Center (DGRC) anad the entry clone encoding the full-length *dNek2* open reading frame was generated using the Gateway System (Thermo Fisher Scientific). All the expression constructs used were generated by the LR reaction with suitable destination vectors following the manual provided by the manufacturer. The dNek2 mutant constructs were generated by

QuickChange Site-Directed Mutagenesis Kit (Agilent Technologies) using the dNek2 entry clone as a template. To generate the D-box mutant forms of dNek2, the first and forth amino acids in the five D-box consensus sequences (RxxL) were mutated to alanine. The single D-box mutants were generated by mutating one D-box at a time: dNek2-1D1 (position 277-280), dNek2-1D2 (position 355-358), dNek2-1D3 (position 634-637), dNek2-1D4 (position 695-698) and dNek2-1D5 (position 715-718). dNek2-5D was generated by sequential mutation of all D-box motifs. dNek2-MR was generated by mutating the first amino acids of the MR motif in position 714-715 to alanine. The kinase dead mutant, dNek2-K48M, was generated by mutating the lysine at position 48 to methionine (Schertel et al., 2013). The dNek2 constructs used for the *in vitro* destruction assays were generated by recombining the dNek2 entry clones with the destination vector pET23b. All DNA sequences were confirmed by DNA sequencing service by Source Bioscience.
The following primers were used:
(For generation of the gateway entry clone of dNek2)
attB-F: GGGGACAAGTTTGTACAAAAAAGCAGGCTTCATGAGCGGAGAGGAATCTGC
stop attB-R: GGGGACCACTTTGTACAAGAAAGCTGGGTCTCAGATCACCATCTGGTCGC
nostop attB-R: GGGGACCACTTTGTACAAGAAAGCTGGGTCGATCACCATCTGGTCGCGCG
(For the generation of the mutant forms of dNek2)
1Dm-F: GCATCGAGGTTATCATTGCGCATCCTGCGGTGGTGCGTAATATTAGC
1Dm-R: TAATATTACGCACCACCGCAGGATGCGCAATGATAACCTCGATGCGT
2Dm-F: TGTTTACTCCAGATTTGGCAAGTGAAGCCTTCTACTCCGCCAAGCGC
2Dm-R:GCTTGGCGGAGTAGAAGTCTTCACTTGCCAAATCTGGAGTAAACACA
3Dm-F:CTGCAACTCGCGTGCAGGCACCTCCTGCGGCCGTCAAGCCACCGATC
3Dm-R:TCGGTGGCTTGACGGCCGCAGGAGGTGCCTGCACGCGAGTTGCAGCT
4Dm-F: GACCCGGAGGAGCACGTGCCCAGCATGCGCGGCAATCGGTGAGGGAA
4Dm-R: CCCTCACCGATTGCCGCGCATGCTGGGCACGTGCTCCTCCGGGTCCA
5Dm-F: TGCAGCGGAACCGCATGGCGAGGTCTGCGGTGGTGGCCACTGGCCAT
5Dm-R: GGCCAGTGGCCACCACCGCAGACCTCGCCATGCGGTTCCGCTGCAGC
MR-F: CGCTGCAGCGGAACCGCGCGGCGAGGTCTTTGGTGGTGGCC
MR-R: CCACCACCAAAGACCTCGCCGCGCGGTTCCGCTGCAGCGTG
K48M-F: GTGAATTGTTCGCCTGGATGGGAATGAACTACGATGAAC
K48M-R: TCATCGTAGTTCATTCCCATCCAGGCGAACAATTCACCG

Cell culture, DNA and dsRNA transfection

Drosophila D.mel-2 cells (Life Technologies) were grown at 25°C in Express Five SFM medium (Life Technologies) supplemented by 2 mM L-glutamine and 1% Penicillin-Streptomycin. DNA transfections were carried out using FuGENE-HD (Promega) according to the manufacturer's instructions. Stably-expressing cell lines were produced by co-transfecting expression plasmids with pCoBlast (Life Technologies) and selecting transgenic cells by regular addition of Blasticidin (Life Technologies) to the culture medium at a final concentration of 30 µg/ml. For RNAi experiments, double-stranded RNA (dsRNA) was produced using the T7RiboMAX Express RNAi System (Promega). dsRNA transfection was carried out in 6 well plates, using 10 µg dsRNA per 1x106 cells with Transfast transfection reagent (Promega) according to the manufacturer's instructions. Two rounds of dsRNA transfection were performed to efficiently deplete endogenous Fzr in *D.mel-2* cells, whereas one round of dsRNA was performed to deplete Apc4.
The following primers were used to generate generate dsRNA.
kanR-F: TAATACGACTCACTATAGGGAGAGACAATCTATCGCTTGTATG
kanR-R: TAATACGACTCACTATAGGGAGAGGAATCGAATGCAACCGGCGC
Fzr-F: TAATACGACTCACTATAGGGAGAATGTTTAGTCCCGAGTAC
Fzr-R: TAATACGACTCACTATAGGGAGACGCTCTGCAGGGTATGAA
Apc4-F: TAATACGACTCACTATAGGGAGAATGGCACAAACGAGCTCC
Apc4-R: TAATACGACTCACTATAGGGAGACGCATTATCACCACCAGA

Preparation of recombinant *Drosophila* Fzr and *S. pombe* Mes1 protein

The recombinant *S. pombe* Mes1 protein was purified as previously described (Kimata et al., 2008) and used at the final concentration of 2.0 µM as an APC/C^{Fzy} inhibitor in the mitotic destruction assays. The recombinant His tag fused *Drosophila* Fzr protein was expressed in Hi Five cells (Thermo Fisher) at Baculovirus Facility at University of Cambridge, purified using Ni-NTA agarose (QIAGEN) and was used at the final concentration of 172 nM in interphase extracts.

Supplemental References

Bischof, J., Bjorklund, M., Furger, E., Schertel, C., Taipale, J., and Basler, K. (2013). A versatile platform for creating a comprehensive UAS-ORFeome library in Drosophila. Development *140*, 2434-2442.

Duncan, D.M., Burgess, E.A., and Duncan, I. (1998). Control of distal antennal identity and tarsal development in Drosophila by spineless-aristapedia, a homolog of the mammalian dioxin receptor. Genes & development *12*, 1290-1303.

Huang, J.Y., and Raff, J.W. (2002). The dynamic localisation of the Drosophila APC/C: evidence for the existence of multiple complexes that perform distinct functions and are differentially localised. Journal of cell science *115*, 2847-2856.

Kimata, Y., Trickey, M., Izawa, D., Gannon, J., Yamamoto, M., and Yamano, H. (2008). A mutual inhibition between APC/C and its substrate Mes1 required for meiotic progression in fission yeast. Dev Cell *14*, 446-454.

Lane, M.E., Sauer, K., Wallace, K., Jan, Y.N., Lehner, C.F., and Vaessin, H. (1996). Dacapo, a cyclin-dependent kinase inhibitor, stops cell proliferation during Drosophila development. Cell *87*, 1225-1235.

Nellen, D., Burke, R., Struhl, G., and Basler, K. (1996). Direct and long-range action of a DPP morphogen gradient. Cell *85*, 357-368.

Olson, E.R., Pancratov, R., Chatterjee, S.S., Changkakoty, B., Pervaiz, Z., and DasGupta, R. (2011). Yan, an ETS-domain transcription factor, negatively modulates the Wingless pathway in the Drosophila eye. EMBO reports *12*, 1047-1054.

Schertel, C., Huang, D., Bjorklund, M., Bischof, J., Yin, D., Li, R., Wu, Y., Zeng, R., Wu, J., Taipale, J., *et al.* (2013). Systematic screening of a Drosophila ORF library in vivo uncovers Wnt/Wg pathway components. Dev Cell *25*, 207-219.

Tavsanli, B.C., Ostrin, E.J., Burgess, H.K., Middlebrooks, B.W., Pham, T.A., and Mardon, G. (2004). Structure-function analysis of the Drosophila retinal determination protein Dachshund. Dev Biol *272*, 231-247.

Xu, T., and Rubin, G.M. (1993). Analysis of genetic mosaics in developing and adult Drosophila tissues. Development *117*, 1223-1237.
