# Supplementary figures and images for: The APC/C Coordinates Retinal Differentiation with G1 Arrest through the Nek2-Dependent Modulation of Wingless Signaling
Source: Dev Cell. 2017 Jan 9;40(1):67–80. doi: 10.1016/j.devcel.2016.12.005 (PMC5225405; doi:10.1016/j.devcel.2016.12.005)

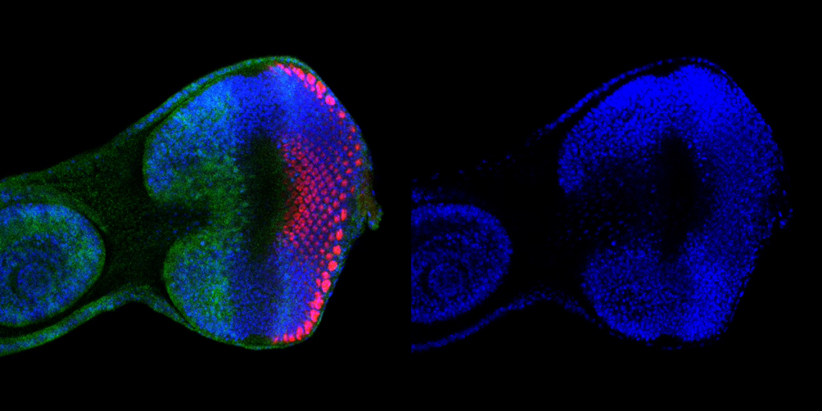

Supplement: Movie S1. Z-Section Animation to Show the Ordered Epithelial Structure of the Control Eye Imaginal Disc, Related to Figure 1 [file mmc2.jpg]

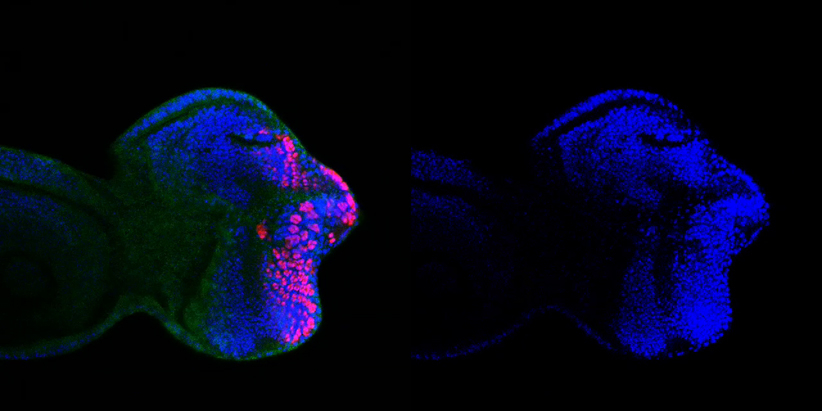

Supplement: Movie S2. Z-Section Animation to Show the Epithelial Structure of the ey>cdc16RNAi1 Eye Imaginal Disc, Related to Figure 1 [file mmc3.jpg]

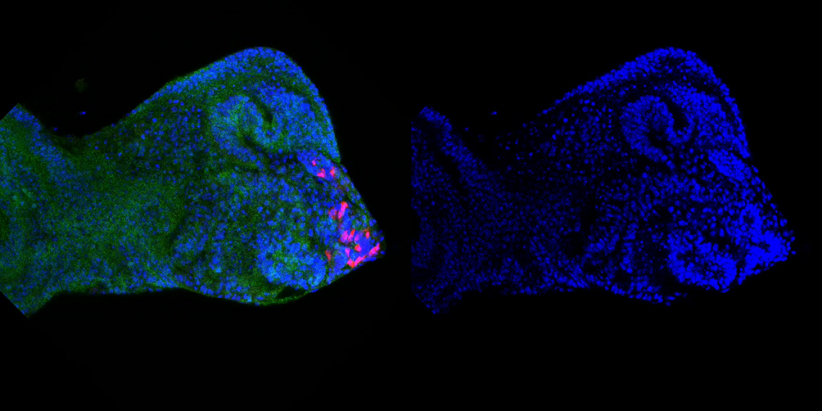

Supplement: Movie S3. Z-Section Animation to Show the Epithelial Structure of the ey>cdc16RNAi1:P35 Eye Imaginal Disc, Related to Figure 1 [file mmc4.jpg]
